# Effects of Micro/Nanoplastics on Crop Physiological Responses and Soil Environment: A Meta-Analysis

**DOI:** 10.3390/toxics14100860

**Published:** 2026-09-28

**Authors:** Aaron Ohene Boanor, Rose Nimoh Serwaa, Jin Hee Park, Jwakyung Sung

**Affiliations:** 1 Department of Crop Science, College of Agriculture, Life and Environment Sciences, Chungbuk National University, Cheongju-si 28666, Chungcheongbuk-do, Republic of Korea; aaronboanor05@chungbuk.ac.kr (A.O.B.); roseserwaanimoh47@chungbuk.ac.kr (R.N.S.); 2 Department of Environmental and Biological Chemistry, College of Agriculture, Life and Environment Sciences, Chungbuk National University, Cheongju-si 28666, Chungcheongbuk-do, Republic of Korea

**Keywords:** microplastic, nanoplastic, nitrogen use efficiency, crop biomass, soil pH, agricultural soils, plant–soil interactions

## Abstract

The increasing accumulation of plastics in agricultural soils poses a threat to soil ecosystems, plants and human life. Under environmental conditions, the accumulation leads to the formation of more reactive forms of plastics such as micro (<5 mm) and nano (<1 µm) plastics. The additives of these plastics are able to leach into the soil and disrupt soil biological, physical and chemical properties as well as affecting plant physiological activities. Given the varying findings across existing studies, this comprehensive meta-analysis helps to synchronize the different results and identify key drivers such as polymer type, shape, particle size, dose, exposure duration, and soil pH. A total of 50 studies with 478 observations were used for this meta-analysis. Overall, exposure of MPs/NPs significantly reduced biomass and NUE by 10.9%, while having only a minor effect on root-to-shoot ratio. Fibers, granular particles, and polyvinyl chloride (PVC), due to their additives, showed the strongest negative impacts on plant performance. PVC produced the greatest reduction in biomass (−24.03%, 95% CI: −67.9–20.7, *). Smaller particles (−9.63%, 95% CI: −80–86.6, ***), higher concentrations (−19.77%, 95% CI: −73.9–73.2, ***), longer exposure periods (−12.15%, 95% CI: −73.9–66.7, ***), and acidic growth media conditions (−12.63%, 95% CI: −80–40.8, ***) further intensified these effects on the biomass and NUE. The findings indicate that MPs and NPs can impair crop productivity by disrupting soil conditions, nutrient availability, and plant physiological processes, highlighting a growing challenge for sustainable agricultural production and soil health. Although advanced analytical techniques are developed, the analytical methods need improvements in the future and more on-field and long-term research studies investigating MP/NP effects on soil and plant ecosystems are required.

## 1. Introduction

The global production of plastics increased to about 413.8 million metric tons in 2024 [1]. Plastics are produced by condensing and adding monomers and additives under a particular temperature [2]. Common examples of these produced plastics are polyvinyl chloride (PVC), polyethylene (PE), low- and high-density polyethylene (LDPE and HDPE), polyethylene terephthalate (PET) and polypropylene (PP) [3]. Additives also combined with polymers and monomers for plastic production include ultra-violet (UV) and thermal stabilizers, colorants, matting agents, opacifiers, luster additives, plasticizers (such as phthalates, diethyl hexyl phthalate, diisononyl ester, citrates, bisphenol A (BPA), adipates) and polymerics [4]. These produced plastics were adopted rapidly for usage due to their flexibility, durability and feasible workability [5]. Some of the uses of plastics include packaging products, pipes and construction materials, household items and agricultural products and materials such as plastic mulch and irrigation lines [6]. Following their use, significant amounts of plastic waste accumulate in soil, which is a major sink, through the agricultural applications of sewage sludge, wastewater irrigation and atmospheric deposition [7]. Accumulated plastics are then broken down into micro/nanoplastics through biological processes, chemical degradation and physical abrasion [8].

Microplastics (plastics particles < 5 mm) and nanoplastics (<1 µm) are emerging contaminants in terrestrial ecosystems and agricultural soils as result of continuous anthropogenic inputs [9,10,11]. Microplastics (MPs) are classified under primary sources when they are gained from household plastics, cosmetics, textiles, cleaning products and the chemical industry whereas they are considered secondary when they are retrieved from the fragmentation of larger plastic products [12]. Although the identification of MPs and their impacts in the aquatic ecosystem have had extensive research and reports, whereas impacts on the terrestrial ecosystem is still ongoing.

MPs have been identified in a wide range of polymer types (PE, PP, PS, PVC and PET) and shapes (films, fibers, fragments and granules) with different physical and chemical characteristics [13]. The structural and chemical differences influence how MPs interact with soil–plant systems and exert biological toxicity. For example, due to the contents of stabilizers and plasticizers in PVC, its MPs cause oxidative stress to both plants and soil biota, impair photosynthesis and reduce the general growth of plants [14,15]. In contrast, PE and PP polymer types, due to their physical properties, can exert physical effects such as altering the soil structure and then atrophying plant roots, leading to the disruption of root growth [10]. Additionally, the interaction with soil and plants can be further determined by the size and shape of the MP/NP. For instance, MPs/NPs which are fibrous can entangle plant roots and then distort the architecture of roots whereas the granular shapes can reduce water movement and soil aeration by blocking the soil pores [9,16]. It has been reported that smaller particle sizes of NPs penetrate root tissues and induce cellular toxicity, such as membrane damage and oxidative stress, when they get into direct contact with plant roots [17,18].

Despite the several extensive studies carried out on the impacts of MPs/NPs on plant and soil environments, majority of the studies were conducted under isolated conditions which has led to different results. These studies normally differed in terms of MP/NP shape, type, size, concentration, exposure duration and plant species, making it difficult to compare the findings and draw a conclusive result. Therefore, to obtain more reliable and comprehensive results, it is necessary to employ a meta-analysis to systematically synchronize the existing findings. For example, a meta-analysis on MP dose, size and crop-type interactions in agricultural ecosystems, undertaken by synthesizing data from 37 studies with 2886 observations, indicated that MPs decreased plant biomass significantly, by 13% (95% CI: 7–19%) [19]. Higher doses and smaller MP particle sizes also showed pronounced effects on root activity and biomass. However, the study did not cover other interactions such as nitrogen use efficiency (NUE), exposure duration and growth medium pH.

Therefore, the main aim of this study is to quantitatively synthesize the risk assessment of how MP properties influence root-to-shoot ratio, biomass, and the NUE of crops using a meta-analytic approach. Specifically, this study evaluates the effects of polymer type, particle size, shape, concentration, exposure duration, and growth medium pH to identify the most influential factors affecting crop performance and NUE in agricultural systems.

## 2. Materials and Methods

The study followed the Preferred Reporting Items for Systematic Reviews and Meta-Analyses (PRISMA) (Figure 1, Supplementary File (PRISMA Checklist)) guidelines to ensure a transparent, reproducible, and unbiased selection of relevant studies [20]. A review protocol was prepared before conducting the searches but was not prospectively registered for this agriculture-focused meta-analysis, therefore there is no appropriate registry available. At least three authors were involved in the independent conduction of literature search, study screening, data extraction and quality assessment.

### 2.1. Literature Search

A comprehensive literature search was performed using three major scientific databases thus; Web of Science, Scopus, and Google Scholar studies published from 2013 to 2025 were included in the analysis. The search strategy was developed using combinations of keywords and Boolean operators (AND, OR). The main search terms included “microplastics AND crops”, “microplastics AND biomass”, “microplastics AND nitrogen use efficiency”, “nanoplastics AND plant growth”, “microplastics AND root to shoot ratio”, “microplastics AND soil pH”, and “microplastics AND agricultural systems”. Additional studies were identified manually from the reference lists of review articles and relevant published papers. A total of 50 studies with a total effect size of 478 observations were used in this meta-analysis (Figure 1, Table S1).

Related research articles were downloaded from the onset and vital information needed for the meta-analysis study were recorded using a Microsoft excel spreadsheet. The information recorded were; study ID, author name, DOI link for the article, publishing year, location of the experiment (longitude and latitude), experimental system (lab, greenhouse or field), crop name, plant type (e.g., cereal/legume), MP polymer type (PE, PP, PVC, PES, biodegradable and PS), shape (fibers, fragments, films, beads, foam, pellets or granular), particle size and dose, exposure duration, pH (before and after experiment) and type of growth medium (soil), fertilizer type and amount used in kg/ha (conversion was done for grams to kg/ha), NUE (treatment and control) and conversion for grams to kg, biomass (conversion was done for grams to kg/ha), root length (mm), plant height (cm) and then root-to-shoot ratio (Table 1).

### 2.2. Data Extraction

#### 2.2.1. Studies Were Included if

- Control treatment was compared with MP or NP exposure.
- Studies were conducted in soil-based crop production systems.
- At least one target response variable was reported: biomass, root-to-shoot ratio and nitrogen use efficiency (NUE).
- Sufficient statistical data for analysis was provided: mean values, standard error (SE) and sample size (n).

#### 2.2.2. Studies Were Excluded if They Met the Following Criteria (Table S2)

- Duplicate studies were identified and removed before screening.
- Titles and abstracts were screened to remove irrelevant studies.
- Appropriate control comparison was lacking.
- Studies comprised reviews, non-peer reviewed reports, book chapters, or conference paper.
- Reported solely on modeling and simulation results.
- Studies unrelated to agricultural crops, aquatic ecosystem studies, or studies not written in English.

Relevant information was extracted from each selected study. Crop-related variables; crop species; biomass; root-to-shoot ratio and NUE. For MP-related variables; polymer type (PE, PP, PVC, PS, PET, biodegradable plastics); particle size (nano, small, medium, large); shape (films, fibers, fragments, granular); concentration (dose) and exposure duration. Environmental variables used were soil pH (growth medium pH) and experimental conditions. Microsoft Office Excel was used to organize the dataset whereas R Studio version 4.4.1 for windows 10^+^ was used to run the dataset [21,22].

### 2.3. Meta-Analysis

Meta-analysis was used because individual studies examine the effects of MPs and NPs on crop biomass, root-to-shoot ratio, NUE on cereal and legume crops often report variables results and, in some cases, contradicting outcomes. By quantitatively synthesizing the data across multiple independent experiments, it increases the statistical power and decrease uncertainty associated with small sample size. Heterogeneity among studies was assessed using Cochran’s Q statistics and quantified using the I^2^ index and between-study variance (τ^2^) (Tables S3–S9) [23]. Multiple linear regression was conducted to determine whether variation in root-to-shoot ratio, biomass, and NUE could be explained by major MP characteristics, including dose, exposure duration, particle size, shape, and polymer type. The multivariable moderator analysis showed that MP characteristics were differently associated with the three plant responses after adjustment for the other moderators, thus root-to-shoot ratio, biomass, and NUE were used as response variables, with dose, exposure duration, particle size, shape, and polymer-type used as predictor variables. The relative importance of MP characteristics on crop responses was determined by grouped permutation importance (ΔMSE) of particle size, exposure duration, dose, shape, and polymer type for (a) root-to-shoot ratio, (b) biomass, and (c) nitrogen use efficiency (NUE). Error bars indicate variability in importance estimates, while study-level five-fold CV R^2^ indicates model predictive performance. Adjusted regression coefficients and their confidence intervals show the direction and magnitude of the relationships between particle size, dose, exposure duration, shape, and polymer type and the LnRR of (a) root-to-shoot ratio, (b) biomass, and (c) nitrogen use efficiency (NUE). The vertical zero line represents no effect, with positive and negative coefficients indicating increases and decreases in the respective crop responses, respectively.

#### 2.3.1. Effect Size

The effect size for this meta-analysis was determined using the log response ratio (LnRR), which is a commonly applied metric in ecological and agricultural meta-analyses for comparing treatment effects relative to the control conversion. The effect of MPs/NPs on biomass, root-to-shoot ratio, and NUE was expressed as an increase or decrease relative to the control treatment.

(1) $$InRRi=In\left(\frac{\bar{X_{ti}}}{{\bar{X}}_{ci}}\right),$$

where X_ti_ represents the mean value of the treatment group exposed to MPs/NPs and X_ci_ represents the mean value of the control group.

The LnRR indicates both the direction and magnitude of the treatment effect:

LnRR < 0 indicates that MPs/NPs caused a reduction in the measured variable.

LnRR > 0 indicates that MPs/NPs increased the measured variable.

LnRR = 0 indicates no effect compared with the control.

The variance of each effect size (Vi) was estimated as:

(2) $$V_{i}=\frac{1}{n_{ti}}+\frac{1}{n_{Ci}},$$

where n_ti_ and n_ci_ are the sample size of the treatment and controls groups respectively.

#### 2.3.2. Percentage Response

To facilitate interpretation, LnRR was converted to percentage response as:

(3) $$\mathrm{Percentage}\;response=(e^{lnRR++}-1)\times 100)$$

Negative value indicates a percent decrease in the measured variable due to the effects of MPs/NPs, whereas a positive value indicates a percentage increase. InRR was selected due to its scale independence and interpretability, while Hedges’ g was included to account for small bias and allow standardized comparisons across studies.

The weighted mean effect size across studies (InRR_++_) was estimated using

(4) $${\mathrm{InRR}}_{++}=\frac{\sum_{i=1}^{k}w_{i}\;\mathrm{LnRRi}}{\sum_{i=1}^{k}w_{\times i}},\;w_{\times i}=\frac{1}{vi+\tau 2},$$

The 95% confidence interval (CI) around the pooled effect size was calculated as:

(5) $$95\%\;\mathrm{CI}={\mathrm{LnRR}}_{++}\pm 1.96\times \mathrm{SE}\;(\mathrm{SE}{\mathrm{LnRR}}_{++}),$$

where *k* is the number of independent effect size included in the analysis and *w_×i_* is the random effects weight assigned to the *ith* effect size.

The standard error of the pooled effect size was calculated as

(6) $$SE({\mathrm{LnRR}}_{++})\sqrt{\frac{1}{\sum_{i=1}^{k}w_{\times i}}},$$

A random-effects model was used because the included studies differed in crop species, soil conditions, MP properties, and experimental design. This model accounts for both within study and between study variation. pooled effect sizes were estimated using a random effect model. Between-study variance (**τ2**) was estimated using the restricted maximum likelihood (REML) method implemented with the *rma* and the metafor package in R version (R studio 4.2.3).

Subgroup analysis was also conducted to evaluate the influence of polymer type, particle size, particle shape, dose, exposure duration and soil pH. This helped identify the most influential MP properties affecting crop performance. Meta regression was performed to assess continuous relationships such as the dose–response relationship, size-dependent effects and exposure duration effects to explain the variability among studies. These analyses were used to determine the reliability and robustness of the meta-analysis results.

#### 2.3.3. Nutrient Use Efficiency

Nutrient Use Efficiency was evaluated using the partial factor of nitrogen productivity defined as

(7) $$NUE=\frac{Y}{Fapplied},$$

where Y = yield, F applied represents the uniform fertilizer (nitrogen fertilizer or nutrient) rate applied in both control and treatment studies. All yields for fertilizer were calculated, standardized to common units prior to calculation.

#### 2.3.4. Statistical Analysis

All analyses and visualizations were conducted in R version (R studio 4.2.3) using REML, vegan, metafor, linkET, and ggplot2 packages. Root-to-shoot ratio indicates changes in plant carbon allocation and growth strategy. Biomass response indicates the direct indicator of plant growth performance and NUE indicates how effectively plants utilize nitrogen for biomass/yield production. The box plot shows how the individual observations are distributed within the percentage response scale. *Y*-axis, which is the percentage response, shows the magnitude and direction of effects, whereas the *X*-axis is the explanatory or moderating variable affecting the three plant traits as response variables. For example, +50% response on the *Y*-axis means the response is 50% higher than the control; however, 0% dashed lines mean no effect reference and the open circles also mean extreme observations. The lower edge of the box is the first quartile (Q1), middle line is the median (Q2) and upper edge box is the third quartile (Q3), whereby the interquartile range (IQR) represents the central 50% of observations. The whiskers indicate the spread of the main body of observations and the diamond inside the box represents the mean percentage responses. N represents the total number of effect-size observations, whereas n represents the number of observations within each microplastic shape category. In almost all of the Figures 3–8, boxes and whiskers extended considerably into the negative and positive regions because the average effects were negative as well as each individual observation, showing a substantial variability.

#### 2.3.5. Sensitivity Analysis and Publication Bias Quality Assessment

To ensure the reliability and validity of the included studies, a publication bias was assessed using funnel plot analysis and the trim and fill method. The funnel plot before and after trim and fill correction showed a generally symmetrical distribution of studies around the overall mean effect size. This suggested that publication bias was relatively low and that the overall conclusions of the study was not influenced substantially. Typically, in a funnel plot, studies with larger standard errors scatter widely at the bottom of the graph while studies with greater precision cluster at the top near the pooled effect size. A symmetrical inverted funnel is formed when publication bias is minimal [24]. Trim and fill method estimates the number of missing or unpublished studies required to produce a symmetrical funnel distribution and recalculates the overall effect size accordingly [25]. Before trim and fill correction, a slight asymmetry toward negative effect size was observed. This is because, in environmental pollution studies, experiments which report harmful effects are often published more than studies which show neutral responses [26]. In this case, MPs and NPs are increasingly recognized as emerging contaminants, therefore, studies demonstrating negative impacts on crops and soil systems would receive greater research and publication attention. In contrast, the trim and fill analysis showed a minor difference between the corrected and uncorrected funnel plots. This means that any potentially missing studies had only a small influence on the pooled effect size. Additionally, the similarity between the two plots suggests that the negative effects observed for biomass, NUE, and root-related traits are robust and unlikely to be driven solely by selective publication. Even after accounting for the potentially missing studies, the overall trends remained negative, indicating that MPs/NPs affect crop performance consistently across different studies and environmental conditions. This therefore makes the confidence in the overall findings of this meta-analysis stronger. For future research to be continued to improve experimental standards, more environmentally realistic concentrations of MPs may be used, more long-term field experiments should be conducted, complete statistical information should be reported and nonsignificant findings then reported together with significant ones. These may help reduce uncertainty and strengthen the future ecological risk assessment of MPs and NPs in agricultural soil ecosystems.

## 3. Results

### 3.1. Global Distribution of Studies on MPs and Their Characteristics

Figure 2 shows the spatial distribution of studies included in this meta-analysis. The figure shows a strong geographic bias towards Asia (especially China) and Europe, with fewer related studies from Africa and South America. Several studies on MPs in agricultural soils are indicated to be from Asia compared with the other continents [27,28]. This uneven distribution therefore reflects the differences in research capacity and the intense usage of agricultural plastic across the regions. Across the studies, MPs varied substantially among their shape, polymer type, particle size, concentration (dose), and exposure of duration. Considering MP/NP shapes, the most common shapes usually used for studies are fibers, films, fragments, and granular forms [28]. Films and fibers appeared more frequently due to their widespread occurrence in the environment because of their higher persistence and mobility [29,30]. Beads, pellets, fragments and foams, on the other hand, are not abundant due to their low spread in the environment [31]. The distribution of polymer type showed that PE, PP, PS, polyester/polyamide, and PVC are the most frequently investigated polymer types [4]. PE and PP dominated the distribution, indicating their widespread usage in agricultural products such as irrigation pipes, packaging materials and mulching films. The geographic spread of polymer types suggests that agricultural practices strongly influence the types of MPs entering the soil.

Particle size distribution showed a dominance of small-sized MP (1–100 µm), followed by medium-sized (100–1000 µm) and lastly, fewer nano-sized particles (<1 µm). Although detection and quantification of NPs remains technically challenging, smaller particles are more abundant because of the fragmentation of lager plastics [12]. In the dose distribution, although some high-dose experiments were included to explore the toxicity threshold, more studies within low to medium concentrations were used. Studies clustering around medium doses suggest the most relevant dose for understanding the real-world impacts of MPs in agriculture [19].

Exposure duration distribution varied widely, with most studies within short (≤30 days) and medium (30–90 days) exposure durations. However, fewer studies investigated long-term exposure duration (>90 days). This means that most experimental studies were often limited by time, which makes long-term accumulation underestimated. Longer exposure durations may depict real impacts of MPs due to cumulative buildup in the soil, progressive soil structure changes, disruption of soil microbes and the increased likelihood of uptake by plants [32]. The overall result of the global dataset suggests that MP effects in agriculture are highly driven by the combination of physical characteristics (size and shape), chemical properties (polymer type) and environmental conditions (dose and exposure duration). However, there is currently an uneven and limited geographical representation of long-term and NP studies. Therefore, future research should prioritize long-term field experiments, improved detection of NPs and broader coverage of MP and NP characteristics.

### 3.2. Impact of MP Shape on Crop Physiology

(Figure 3, Table S3) presents three boxplots showing the percentage responses of root-to-shoot ratio, biomass, and NUE under exposure to different MP shapes (films, fibers, granular particles, and fragments). Each boxplot summarizes the distribution of effect sizes derived from multiple studies and provides insight into both the magnitude and variability of MP-induced responses. The root-to-shoot ratio was not significantly affected by any MP shape, although fibers produced the largest reduction (−2.24%, 95% CI: −90.1–111.3, ns), followed by films (5.30%, 95% CI: −40–75, **), fragments (0.61%, 95% CI: −33.3–43.9, ns), and granular particles (0.75%, 95% CI: −48–42.2, ns). The overall effect size for root-to-shoot ratio was small and statistically non-significant (N = 396, effect size = 1.03, ns) (Figure 3). The lack of significant effects suggests that MP exposure did not substantially alter biomass allocation between belowground and aboveground plant organs.

In contrast to root-to-shoot ratio, biomass was significantly reduced by all MP shapes. Fibers caused the largest decline in biomass (−8.99%, 95% CI:−68.8–90.5, ***), followed by films (−4.43%, 95% CI: −73.9–86.6, *), granular particles (−7.85%, 95% CI: −80–100, *), and fragments (−5.43%, 95% CI: −67.9–100, ns). The overall biomass response was significantly negative (N = 379, effect size = −6.53%, ***) (Figure 3). Nitrogen use efficiency was also significantly affected by MP shapes (−6.15%, ***) (Figure 3). Fibers produced the greatest reduction (−10.51%, 95% CI: −68.2–90.5, ***), followed by films (−4.34%, ns), granular particles (−5.52%, ns), and fragments (−2.85%, ns).

### 3.3. Impact of MP Type on Crop Physiology

The polymer type plays a critical role in determining the magnitude of phytotoxic effects, with substantial variation observed among PE, PP, PES, PS, PVC, Bio, and other polymer categories (Figure 4, Table S4).

While root-to-shoot ratio remained relatively unaffected by polymer type, biomass and NUE were significantly reduced, indicating that MP-induced stress primarily affected plant growth and nutrient utilization rather than biomass allocation. Polypropylene was the only polymer associated with a significant increase in root-to-shoot ratio (10.79%, 95% CI: −9.10–56, ***). The overall effect across all polymer types was small and non-significant (1.03%, ns), suggesting that MP exposure did not substantially alter carbon allocation between belowground and aboveground tissues (Figure 4).

Biomass was significantly reduced by several polymer types, confirming that plant growth is highly sensitive to MP contamination. Among the polymers examined, PVC produced the greatest reduction in biomass (−24.03%, 95% CI: −67.9–20.7, *), followed by Bio (−13.11%, 95% CI: −25–2.78, ***), PES (−9.69%, 95% CI: −60–66.7, ns), PE (−8.11%, 95% CI: −54.1–39.1, ***), and PS (−4.71%, 95% CI: −60–66.7, ns). Polypropylene caused the smallest reduction (−3.05%, 95% CI: −66.7–36.7, ns) (Figure 4).

Nitrogen use efficiency followed a pattern similar to biomass, indicating that reductions in plant growth were closely linked to disruptions in nitrogen acquisition and utilization. Polyvinyl chloride again produced the strongest negative response (−24.30%, 95% CI: −67.9–20, *), followed by Bio (−11.80%, 95% CI: −25–2.78, ***), PES (−9.69%, 95% CI: −73–66.7, *), PE (−8.06%, 95% CI: −70.3–100, **), and PS (−5.86%, 95% CI: −80–90.5, ns). Polypropylene remained the least damaging polymer type (−3.05%, 95% CI: −66.7–50, ns) (Figure 4).

### 3.4. Impact of MP Size on Crop Physiology

Particle size is one of the most important determinants of MP toxicity because it influences particle mobility, root penetration, cellular uptake, translocation, and interactions with soil properties. Microplastic size significantly influenced biomass and NUE, whereas root-to-shoot ratio remained largely unaffected (Figure 5, Table S5).

The results indicate that smaller particles exerted stronger inhibitory effects on crop growth and nutrient utilization than larger particles, highlighting the importance of particle size as a key driver of phytotoxicity.

Root-to-shoot ratio showed relatively small and statistically non-significant responses across all particle size classes. Nano-sized particles increased root-to-shoot ratio by 9.90% (95% CI: −33.9–43.7, ns), medium-sized particles increased it by 3.26% (95% CI: −48–69.8, ns) and large particles increased it by 3.80% (95% CI: −30–42.1, ns), whereas small particles reduced it slightly by 1.01% (95% CI: −90.1–111, ns). Despite these variations, none of the responses were statistically significant (Figure 5).

Biomass was significantly affected by particle size. Small particles (1–100 μm) caused the greatest reduction in biomass (−9.63%, 95% CI: −80–86.6, ***), followed by medium-sized particles (−9.95%, 95% CI: −73.9–66.7, ***). Nano-sized particles produced a smaller reduction (−2.45%, 95% CI: −46–90.1, ns), while large particles resulted in a non-significant decline (−5.59%, 95% CI: −66.7–100, ns) (Figure 5).

The pattern observed for NUE closely mirrored that of biomass. Small particles produced the largest reduction (−9.88%, 95% CI: −80–86.6, ***), followed by medium-sized particles (−9.95%, 95% CI: −73.9–66.7, ***), while nano-sized and large particles caused smaller, non-significant reductions (−1.05%, and −5.59%, respectively) (Figure 5).

### 3.5. Impact of MP Dose on Crop Physiology

Microplastic concentration is a critical factor determining the extent of phytotoxic effects because increasing particle abundance elevates the probability of interactions between plastics, soil constituents, microorganisms, and plant tissues. Microplastic dose significantly influenced biomass and NUE, whereas root-to-shoot ratio remained largely unaffected (Figure 6, Table S6).

The results indicate that increasing exposure concentration intensified adverse effects on plant productivity and nutrient utilization, highlighting dose-dependent toxicity in crop systems.

Root-to-shoot ratio was relatively stable across all concentration classes and did not exhibit statistically significant responses. High doses (>1) resulted in a positive response of (8.09%, 95% CI: −66.1–75) whereas medium, low, and very low doses produced small negative or neutral responses ranging from −0.27% to −1.22% (Figure 6).

Biomass was strongly influenced by MP concentration. High doses (>1) caused the greatest reduction in biomass (−19.77%, 95% CI: −73.9–73.2, ***), followed by low doses (−10.15%, 95% CI: −67.9–100, ns), very low doses (−9.22%, 95% CI: −68.8–59.8, ns), and medium doses (−3.55%, 95% CI: −80–90.5, *) (Figure 6).

Nitrogen use efficiency exhibited a pattern similar to biomass. High concentrations resulted in the greatest reduction (−19.82%, 95% CI: −73.9–73.2, **), whereas medium concentrations produced the smallest decline (−3.54%, 95% CI: −80–90.5, *). Low and very low concentrations reduced NUE by 7.33% and 4.52%, respectively, although these responses were not statistically significant (Figure 6).

### 3.6. Impact of MP Exposure Duration on Crop Physiology

The duration of exposure is a critical determinant of contaminant toxicity because biological responses often evolve over time. Short-term exposure may induce immediate physiological disturbances, whereas prolonged exposure can lead to cumulative effects resulting from sustained interactions between MPs, soil processes, and plant metabolic functions. The exposure duration significantly influenced biomass and NUE but had limited effects on root-to-shoot ratio (Figure 7, Table S7).

Notably, long-term exposure (>90 days) caused the greatest reductions in biomass and NUE, suggesting that chronic exposure amplifies the adverse effects of MPs on crop performance.

Root-to-shoot ratio was not significantly affected by exposure duration. Short-term exposure (<30 days) increased root-to-shoot ratio by 1.25% (95% CI: −90.1–111), medium-term exposure (30–90 days) increased it by 3.07% (95% CI: −69.2–75), whereas long-term exposure (>90 days) resulted in a slight decline of 2.75% (95% CI: −48–66.7) (Figure 7).

Biomass responses varied considerably with exposure duration. Long-term exposure produced the largest reduction (−12.15%, 95% CI: −73.9–66.7, ***), followed by short-term exposure (−5.57%, 95% CI: −68.8–100, *), whereas medium-term exposure resulted in a relatively small and non-significant decline (−2.12%, 95% CI: −80–86.6, ns) (Figure 7).

Nitrogen use efficiency exhibited a pattern nearly identical to biomass, with long-term exposure causing the largest reduction (−12.15%,95% CI: −73.9–66.7, ***), followed by short-term exposure (−2.76%, 95% CI: −68.8–100, ns), while medium-term exposure remained statistically non-significant (−2.12%, 95% CI: −69.2–75, ns) (Figure 7).

### 3.7. Influence of Growth Medium pH on MP Toxicity

pH is one of the most influential environmental variables regulating nutrient availability, microbial activity, contaminant mobility, and root physiological processes. Consequently, the MP toxicity may vary considerably across pH conditions because the physicochemical behavior of plastic particles changes with the surrounding chemical environment. The growth medium pH significantly modified the effects of MPs on biomass and NUE, while root-to-shoot ratio remained largely unaffected (Figure 8, Table S8).

Among the pH categories examined, acidic conditions produced the greatest reduction in biomass, whereas slightly alkaline conditions resulted in the strongest decline in NUE.

Root-to-shoot ratio exhibited limited sensitivity to growth medium pH. Acidic and neutral media produced small positive responses of 2.16% (95% CI: −69.2–69.8, ns) and 0.32% (95% CI: −90.1–111, ns) respectively, whereas slightly alkaline conditions generated a modest negative response (−6.21%, 95% CI: −33.9–75, *) (Figure 8). Biomass was significantly reduced under acidic conditions (−12.63%, 95% CI:−80–40.8, ***), whereas neutral and slightly alkaline conditions produced smaller, non-significant reductions of −1.64% (95% CI:−68.8–100, ns) and −3.98% (95% CI:−67.9–100, ns) respectively (Figure 8). Unlike biomass, NUE was most strongly reduced in slightly alkaline conditions (−12.29%, 95% CI:−67.9–71.4, ***), while acidic and neutral media showed smaller and non-significant responses of −4.84% and −4.64%, respectively (Figure 8).

### 3.8. Comprehensive Assessment of MP Impacts on Crop Physiology

Figure 9 (Frequency and distribution curve) has three analytical variables: effect size magnitude and direction indicated by the *X*-axis; the frequency/distribution of observations, indicated by the histogram and the red density curve, respectively; and the overall mean effect and statistical significance, indicated by the dashed line. The effect size (InRR) on the *Y*-axis represents the percentage change scaling between −2 to 1. The figure reports the number of effect size observations (N), the statistical significance (*p*) and the mean percentage response effect (InRR). The gray bars that form the histogram show how frequently different InRR values occur, thus the distribution of individual effect sizes. The width of the histogram is the effect size interval, while the height indicates how frequently observations occur within that interval. The peak of the red density curve indicates where observations are concentrated; for example, biomass had its greatest concentration close to zero but extended towards the negative InRR values, which means many biomass effect sizes are relatively close to zero. The overall result diagram demonstrates that MPs/NPs exert a substantially negative effect on plant biomass and NUE, while producing a comparatively weaker effect on root-to-shoot ratio. The overall pooled effects of sizes revealed a significant reduction in biomass and NUE of approximately −10.9% (*p* < 0.001), whereas the reduction in root-to-shoot ratio was relatively small and statistically nonsignificant (−1.3%, *p* = 0.281). On the frequency distribution for the overall effects, significant shifts are seen in biomass (N = 379, Mean InRR = 0.12, *p* < 0.001) and NUE (N = 328, Mean InRR = 0.12, *p* < 0.001), while a minor insignificant shift is seen in the root-to-shoot ratio (N = 396, Mean InRR = 0.01, *p* = 0.281) frequency distribution (Figure 9, Table S9). This means that MPs/NPs primarily impair physiological and metabolic processes associated with nutrient acquisition and growth rather than drastically altering biomass partitioning between roots and shoots.

The importance of the five variables of Figure 10 (particle size, exposure duration, dose, shape and type) of the metaforest figure were quantified using grouped permutation importance (ΔMSE) for the three response variables thus; root-to-shoot ratio, biomass and NUE. The grouped permutation importance (ΔMSE = mean squared error) measures how far the model’s predictions from the observed values. Therefore, the larger the ΔMSE, the greater the predictive importance of that variable in the model. Vice-versa, a small ΔMSE means relatively little predictive information. The error bars represent the uncertainty or variability around the permutation importance estimate. The horizontal error bars represent the 95% confidence intervals of permutation importance. The CV R^2^ is the five-fold cross validation which represents the model’s out-of-sample predictive performance. For example, R^2^ for biomass was 0.415 (41.5%) which means greater predictive information than that of root-to-shoot ratio. The MetaForest-based permutation importance analysis showed that exposure duration was the most important predictor of biomass and NUE, followed by dose and particle size. For root-to-shoot ratio, particle size had the greatest importance, followed by exposure duration and shape. Polymer type contributed comparatively little to all three responses. The results indicate that exposure duration and particle characteristics may be more influential than polymer type in determining plant responses to MPs. The models showed higher predictive performance for NUE (CV R^2^ = 0.442) and biomass (CV R^2^ = 0.415) than for root-to-shoot ratio (CV R^2^ = 0.143). The near zero mean InRR and the non-significant overall effect indicate that the root-to-shoot ratio was relatively insensitive to MP exposure. Therefore, the relatively low CV R^2^ for root-to-shoot ratio may reflect the limited variability in this response attributable to the selected moderators.

The adjusted coefficient of Figure 11 indicates the expected lnRR changes when a particular predictor changes, while the other predictors in the model are held constant. For example, larger particle size was associated with a more positive lnRR after adjusting for exposure duration, dose, shape, and polymer type. The horizontal line extending to the left and right represents the uncertainty intervals around the adjusted coefficient. Therefore, if the entire interval was above zero, then the association was statistically positive at approximately the 5% level and vice versa. For the categorical variables, granular was the reference category for shape and PE was also the reference category for polymer type. The overall moderator significance, shown by the shape omnibus p and type omnibus p, indicates whether the whole categorical variable significantly explains the variation. For example, although films versus granular may show a positive coefficient, if its confidence interval spans zero, this specific comparison should not be interpreted as statistically significant even though the overall shape omnibus test is significant. The multivariable moderator analysis showed that MP characteristics were differently associated with the three plant responses after adjustment for the other moderators. For root-to-shoot ratio, most coefficients were small, although PP showed a positive association relative to PE (0.15). For biomass and NUE, dose and exposure duration were negatively associated with the responses, while film-shaped particles showed positive effects (0.08 and 0.10, respectively). PVC produced the strongest negative association for both biomass (−0.33) and NUE (−0.38), whereas PP and PS showed positive associations. Overall, the results indicate that polymer type and particle shape contributed substantially to variation in biomass and NUE, together with the negative associations observed for dose and exposure duration.

## 4. Discussion

### 4.1. Impact of MP on Root-to-Shoot Ratio

Root-to-shoot ratio is a key indicator of carbon partitioning and resource allocation, reflecting how plants balance investment between nutrient acquisition and photosynthetic structures [33]. Root-to-shoot ratio is often maintained within a relatively narrow range because it reflects a fundamental balance between resource acquisition below ground and carbon assimilation aboveground [34]. Under stress conditions, plants frequently adjust total growth rather than altering allocation patterns dramatically.

The slightly negative response observed in fiber treatments may be associated with the physical interference of fibers within the soil matrix. Fibers can entangle roots and modify pore connectivity, potentially restricting root elongation and altering water movement around roots [35]. However, the observed reductions were insufficient to induce major shifts in biomass partitioning. These findings agree with previous studies reporting that MPs often affect total biomass more strongly than biomass allocation patterns [36].

The increase in root-to-shoot ratio observed under PP exposure may represent an adaptive stress response whereby plants allocate proportionally more biomass to roots to enhance nutrient and water acquisition under unfavorable conditions [37]. Similar increases in root allocation have been reported under nutrient limitation, drought stress, and soil physical disturbances [38]. Polypropylene is generally considered one of the least chemically reactive polymers and contains fewer toxic additives than PVC and some biodegradable plastics [39]. Consequently, plants exposed to PP may maintain normal physiological functioning while adjusting biomass allocation to compensate for reduced resource availability [40]. Nevertheless, the lack of significant effects indicates that most polymer types reduce overall plant growth without substantially altering biomass partitioning patterns.

The slight increase observed in nano-sized particles may indicate a compensatory response in which plants allocate more resources to root development to enhance nutrient and water uptake under MP-induced stress, with similar adaptive responses having been reported under drought, nutrient deficiency, and contaminant exposure [41,42]. The absence of significant changes in the root-to-shoot ratio by dose of MPs suggest that plants maintained proportional biomass allocation despite exposure to varying MP concentrations. This observation indicates that carbon partitioning mechanisms are relatively resilient to concentration-dependent plastic stress. Resource allocation patterns are regulated by complex hormonal networks involving auxins, cytokinin’s, and abscisic acid, which often preserve functional balance between root and shoot development even when total growth is reduced [43,44]. However, the considerable variability among observations suggests that this response was highly species-dependent and influenced by experimental conditions.

The slight decline observed in root-to-shoot ratio under long-term exposure may indicate gradual reductions in root vigor resulting from prolonged stress [45,46]. However, the effect was insufficient to induce substantial shifts in carbon allocation. These findings suggest that MP exposure duration influences overall productivity more strongly than biomass distribution [47]. Plants frequently regulate shoot and root growth in a coordinated manner across a broad pH range, allowing them to maintain functional balance despite changes in soil chemistry [48]. The stronger inhibition of root-to-shoot ratio observed in acidic media may be linked to enhanced surface modification of MPs under low pH conditions.

### 4.2. Impact of MP Type on Crop Biomass

The overall reduction in biomass supports numerous reports showing that MPs interfere with root growth, photosynthesis, nutrient acquisition, and water relations, all of which contribute to lower biomass production [49,50]. Fibrous MPs can become physically intertwined with roots, reducing root penetration, impairing soil–root contact, and disrupting water and nutrient uptake processes [51]. Furthermore, fibers have been shown to alter soil bulk density and pore distribution, leading to changes in aeration and water retention [9]. Granular particles also caused substantial biomass reductions. Their relatively compact shape may obstruct root pores and interfere with root growth by modifying soil aggregation and nutrient diffusion pathways. Previous studies have shown that granular MPs can reduce root development and increase oxidative stress in crops, ultimately reducing biomass accumulation [50].

The strong inhibitory effect of PVC on biomass is consistent with previous studies identifying PVC as one of the most hazardous plastic polymers in terrestrial ecosystems [52,53]. PVC contains numerous additives, including phthalates, stabilizers, lubricants, and heavy-metal-based compounds that may leach into the surrounding soil environment during degradation [54,55]. These compounds can induce oxidative stress, disrupt membrane integrity, inhibit photosynthesis, and impair nutrient uptake. Several studies have reported increased reactive oxygen species (ROS) production and lipid peroxidation in plants exposed to PVC MPs, leading to reduced biomass accumulation [56].

The stronger inhibitory effects of small- and medium-sized MPs may be attributed to their optimal combination of mobility and persistence within the soil environment. Particles within the 1–1000 μm size range can readily accumulate in root zones, modify soil pore structure, and interfere with root–soil interactions [10,51]. Their relatively large surface area also enhances interactions with nutrients, soil colloids, and root surfaces. Small and medium-sized particles clog root pores, alter rhizosphere conditions, and reduce nutrient diffusion toward roots [9]. Furthermore, they may adsorb essential nutrients and decrease their availability for plant uptake. These mechanisms collectively contribute to reductions in plant growth and biomass accumulation. Although NPs possess high mobility and can enter plant tissues, their overall effect on biomass was weaker than that of small particles [57]. One possible explanation is that the dataset contained fewer observations (n = 23) compared with small and medium-sized particles, reducing statistical power. In addition, NPs often produce species-specific responses, with some studies reporting growth inhibition and others reporting negligible effects depending on concentration and surface chemistry. Another explanation is that NPs may move through plant tissues without causing substantial physical disruption to soil structure observed with larger particles [58,59]. Consequently, their effects may be more physiological than structural.

The pronounced inhibition observed at high MP concentrations is likely related to the accumulation of MP particles within the rhizosphere [60]. As particle abundance increases, soil pore spaces become increasingly occupied by plastic materials, creating a physical environment that differs substantially from uncontaminated soils. Another mechanism may involve alterations in rhizosphere chemistry [50]. High concentrations of MPs can modify the adsorption and desorption behavior of nutrients and organic compounds, thereby reducing nutrient accessibility. Once concentrations exceed a critical threshold, compensatory mechanisms become insufficient to offset stress-induced growth reductions.

The greater effect observed during prolonged exposure may be attributed to the progressive aging and weathering of MP particles. As exposure time increases, MPs undergo physical and chemical transformations, including surface cracking, oxidation, and fragmentation [50,61]. These processes increase surface roughness and reactivity, enhancing interactions with plant roots and soil constituents [62]. Microplastics can gradually release residual monomers, additives, and absorbed environmental pollutants over time [63]. Such compensation could involve adjustments in metabolic activity, root turnover, or nutrient acquisition strategies before chronic stress effects become fully established [64].

Acidic environments can accelerate oxidation and weathering processes on plastic surfaces, increasing the formation of oxygen-containing functional groups such as carboxyl and hydroxyl groups [65]. These changes increase the reactivity of MPs and may strengthen their interactions with root surfaces and dissolved compounds. Low pH conditions can also increase the mobility of certain metal ions that are adsorbed onto MP surfaces [66]. Microplastics often act as carriers for trace metals, and acidic conditions may facilitate their desorption into the rhizosphere, increasing plant exposure to potentially harmful substances [67]. This mechanism could contribute to the stronger reductions in biomass observed under acidic conditions. In contrast, the relatively weak responses observed in neutral pH media may reflect a greater chemical stability of both the soil environment and MP particles.

### 4.3. Impact of MPs on Nitrogen Use Efficiency

Because nitrogen acquisition depends heavily on root–soil interactions, any physical impediment to root growth can reduce nitrogen uptake efficiency. Microplastics can also alter soil microbial communities responsible for nitrogen cycling with several studies that have reported that plastic contamination reduces the abundance and activity of nitrifying and denitrifying microorganisms, thereby disrupting nitrogen mineralization and nutrient availability [68,69,70,71]. Reduced microbial activity may decrease the conversion of organic nitrogen into plant available forms, thereby lowering NUE. Additionally, oxidative stress induced by MP exposure can impair root membrane integrity and transporter activity, limiting nitrate and ammonium absorption. Reduced chlorophyll synthesis and photosynthetic performance further constrain nitrogen utilization because photosynthesis provides the carbon skeletons necessary for amino acid production [72,73].

The strong decline in NUE under fiber exposure likely reflects disruptions in both nitrogen uptake and nitrogen assimilation processes. Fibers can alter soil structure, reduce root surface area exploration, and affect microbial communities responsible for nitrogen mineralization and transformation [74]. The reduction in NUE under PVC exposure likely results from multiple interacting mechanisms. First, PVC-induced root damage reduces the ability of plants to absorb nitrate and ammonium from the soil [75]. Additionally, toxic additives released from PVC can impair membrane transport proteins involved in nutrient uptake. Impaired membrane transport damage can also increase oxidative stress which can damage root tissues and reduce nutrient transport efficiency [76]. The significant reduction in NUE observed under biodegradable plastic exposure may be related to changes in microbial decomposition processes [77]. Additionally, in another study, biodegradable PLA-PBAT affected N-partitioning at the early stages of degradation with a lower assimilation of N [78]. This is because, during degradation, the plastic changes chemically and biologically, thereby creating sites which are able to absorb nutrients and also stimulate microorganisms to temporarily immobilize supplied nutrients [79]. PVC consistently produced the strongest negative effects on biomass and NUE, indicating that its chemical composition and additive content contribute substantially to plant toxicity. In contrast, PP exhibited the weakest effects, suggesting that polymer stability and lower additive concentrations reduce phytotoxicity. The close correspondence between reductions in biomass and NUE suggests that impaired nutrient acquisition and utilization are key mechanisms through which MPs reduce plant productivity.

Small and medium-sized particles can reduce root growth and root surface area, limiting the capacity of plants to absorb nitrate and ammonium from the soil [80]. Reduced root exploration decreases access to nutrient-rich microsites and lowers overall nitrogen uptake efficiency. Previous studies have demonstrated that MP contamination can reduce microbial diversity, alter enzyme activities, and disrupt nutrient cycling processes [81,82]. Consequently, the availability of plant-accessible nitrogen may decline, resulting in lower NUE, with MPs frequently inducing oxidative stress, leading to elevated production of reactive oxygen species (ROS) [83,84]. Oxidative damage to root membranes can impair nutrient transporter activity, reducing nitrogen absorption and translocation. Additionally, reduced photosynthetic performance limits the supply of the carbon skeletons required for nitrogen assimilation into amino acids and proteins, further decreasing NUE [72,76]. The results suggest that toxicity generally increased as particle size decreased from large to small MPs. Smaller particles possess a greater specific surface area, which enhances their reactivity and interaction with soil particles, contaminants, and biological tissues, they are also more readily transported within soil pores and can accumulate near root surfaces where they interfere with nutrient and water uptake [85]. In contrast, larger particles have limited mobility and reduced opportunities for direct interaction with roots, their effects are therefore primarily indirect and less severe [86]. As environmental weathering progressively fragments larger plastics into smaller particles, the long-term accumulation of small MPs in agricultural soils may pose increasing risks to crop productivity.

Elevated MP concentrations can reduce the effectiveness of nutrient acquisition and utilization processes by modifying rhizosphere conditions and disrupting root functionality. In soils, nitrate and ammonium move toward roots through mass flow and diffusion [87]. Therefore, high particle densities may also interfere with nutrient diffusion pathways. The accumulation of MPs can create discontinuities within the soil matrix, reducing the efficiency of nutrient transport toward root surfaces [32]. Consequently, plants may absorb less nitrogen despite their presence in the surrounding environment. Another possible explanation is the influence of concentrated MP contamination on microbial-mediated nutrient transformations. High concentrations can alter microbial community composition, affecting nutrient turnover rates and the release of plant-available nitrogen forms [88]. Reduced nutrient availability ultimately decreases NUE and limits biomass accumulation.

Unlike short-term treatments, chronic exposure may affect internal nitrogen metabolism rather than merely reducing nutrient uptake. One possible mechanism involves the disruption of nitrogen assimilation pathways [74]. Long-term exposure to MPs has been associated with alterations in the activities of nitrate reductase, glutamine synthetase, and other enzymes involved in nitrogen metabolism [89]. Reduced enzyme activity limits the conversion of inorganic nitrogen into amino acids and proteins, thereby lowering NUE. Extended exposure may also influence root turnover and root lifespan [62]. Consequently, older root systems may become less effective at supporting nitrogen uptake during later growth stages [90]. Beneficial rhizosphere organisms, including nitrogen-transforming bacteria and plant growth-promoting rhizobacteria, can be negatively affected by prolonged plastic exposure, reducing nutrient availability and plant nutrient use efficiency [91]. The stronger negative responses observed under long-term exposure highlight the importance of considering exposure duration when evaluating MP risks. Over extended periods, plastic particles become increasingly integrated into the soil environment. This integration can influence soil aggregation processes, root–soil contact dynamics, and rhizosphere development. Consequently, adverse effects that may initially appear minor can become amplified over time. Long-term exposure may also coincide with critical developmental stages such as flowering, grain filling, and reproductive growth. Stress occurring during these stages can have disproportionately large effects on biomass accumulation and NUE compared with stress experienced during early vegetative growth [92].

The greater decline in NUE under alkaline conditions may be associated with pH-dependent changes in nitrogen chemistry [93]. In alkaline environments, ammonium-based nitrogen is more susceptible to conversion into gaseous ammonia, reducing the pool of plant-available nitrogen [94]. When combined with MP-induced stress, this reduction in nitrogen availability may further impair NUE. Another possible explanation involves the adsorption behavior of nutrients on MP surfaces. Previous studies have shown that pH can influence the sorption of dissolved compounds onto plastic particles, potentially altering nutrient dynamics within the rhizosphere [95,96]. Alkaline conditions may also reduce the availability of micronutrients such as iron, zinc, manganese, and copper [97]. These elements serve as cofactors for numerous enzymes involved in nitrogen assimilation and protein synthesis. Reduced micronutrient availability may therefore decrease the efficiency with which plants convert absorbed nitrogen into biomass, resulting in lower NUE [88].

The significant reductions in biomass and NUE indicate that MP contamination may have important implications for crop productivity and nutrient management. Lower biomass production directly translates into reduced carbon sequestration and potentially lower yields. Simultaneously, reduced NUE implies that crops require greater fertilizer inputs to achieve the same productivity, thereby increasing production costs and environmental risks associated with nitrogen losses.

Biomass was the most negatively affected in acidic media, whereas NUE was most strongly reduced in slightly alkaline media. This divergence suggests that different mechanisms govern plant growth and nitrogen utilization. Biomass production appears to be more sensitive to pH-induced changes in contaminant behavior and root stress, whereas NUE may be more strongly influenced by pH-dependent nutrient transformations and nutrient availability. The results highlight the complexity of MP–soil interactions and demonstrate that pH not only influences plant physiology directly but also modifies the environmental behavior of plastic particles. The findings indicate that growth medium pH functions as an important moderator of MP toxicity. Changes in pH can alter particle aggregation, surface chemistry, contaminant release, and interactions with soil colloids [98].

### 4.4. Comprehensive Assessment of the Impacts of MPs on Crop Physiology

The narrower distribution and near-zero mean InRR of the root-to-shoot ratio suggest that biomass allocation was relatively stable compared with total biomass production and NUE. The root-to-shoot ratio displayed the weakest relationships with MP characteristics, indicating that biomass allocation is relatively insensitive to variation in particle properties. The absence of strong predictor-response in root-to-shoot relationships suggests that biomass allocation is influenced by compensatory developmental mechanisms operating within the plant [50]. Root and shoot growth are interconnected through whole-plant carbon budgeting, and alterations in one organ are often accompanied by adjustments in the other parts [48]. Consequently, disturbances imposed by MPs may not necessarily produce proportional shifts in biomass partitioning.

The distributions of biomass and NUE were centered below zero and showed extended negative tails, indicating an overall tendency toward negative responses to MPs/NPs. The density distributions centered around the negative InRR values indicates the inhibitory responses which predominated within the dataset. Biomass accumulation reflects the integrated outcome of photosynthesis, nutrient uptake, carbon assimilation and water use efficiency. Therefore, the reductions in biomass suggest that MPs/NPs can disrupt multiple plant physiological processes simultaneously. For example, additives in MPs/NPs can cause the excessive production of reactive oxygen species (ROS) in plant tissues leading to chlorophyll damage and impaired photosynthesis. Reduced photosynthetic activities can directly decrease carbon assimilation, thereby limiting the production of biomass [99]. MPs significantly decreased photosynthetic rate (Pn), stomatal conductance (Gs), transient transpiration rate (Tr), electron transport rate (ETR) and photosystem II (Fv/Fm) but increased intercellular CO_2_ concentration (Ci) in lettuce leaves, indicating that MPs reduced photosynthetic activities [100]. NUE also had a significant reduction and shift because NUE is closely linked to root functioning, microbial nutrient cycling and nutrient transport within plant tissues. NUE was reduced significantly due to the physical blockage in the soil pores by different MP/NP sizes and doses, MP/NP adsorption of nutrients such as ammonium, nitrate and phosphate onto their surfaces and the disruption of microbial activities in the rhizosphere [101].

Biomass showed sensitivity to both exposure duration and dose. The association between exposure duration and biomass suggests that longer exposure may be related to greater biomass reduction. Contaminant concentration and exposure period are frequently manipulated together to evaluate chronic stress responses [46,86,102]. The dominant influence of dose on biomass suggests that concentration remains the primary determinant of growth suppression. As MP abundance increases, the cumulative area occupied by particles within the root zone also increases [62]. This may disrupt root–soil contact efficiency, reducing the effectiveness of water and nutrient acquisition [46]. Unlike previous interpretations involving nutrient limitations or oxidative stress, another possible mechanism involves alterations in root hydraulic continuity. Plant water uptake depends on uninterrupted water films connecting soil particles and roots [46]. High MP concentrations may fragment these pathways and reduce hydraulic conductivity around the rhizosphere, leading to chronic water deficits that ultimately suppress biomass accumulation [38,103]. Smaller particles possess greater particle number densities at equivalent mass concentrations, increasing the probability of interaction with root surfaces and biologically active microsites [47]. Consequently, a given quantity of small particles can exert a stronger influence on plant growth than an equivalent mass of larger particles [33].

The sensitivity of NUE to MP concentration suggests that nitrogen utilization processes are particularly vulnerable to increasing contamination levels. NUE reflects the integration of nitrogen acquisition, assimilation, transport, storage, and remobilization [104]. Disruption of any component can reduce overall efficiency. Nitrogen acquisition depends heavily on communication between plant roots and beneficial microorganisms inhabiting the rhizosphere [105]. Increasing MP loads may alter the spatial organization of microbial habitats and disrupt signaling exchanges involved in nutrient acquisition [64]. Such disturbances could reduce the efficiency with which plants coordinate nitrogen uptake with metabolic demand. Greater physical heterogeneity may create microscale disruptions in nutrient diffusion pathways, thereby reducing NUE [106]. Their reduced mobility and lower surface-area-to-volume ratio may lessen their interactions with rhizosphere processes compared with nano- and microscale particles [107].

Dose exhibited the negative adjusted coefficients for both biomass and NUE, indicating a general tendency toward stronger adverse responses with increasing MP concentrations. As MP loading increases, the cumulative effects on resource acquisition and metabolic functioning become increasingly pronounced [108]. Exposure duration was an important predictor in the MetaForest analysis, while the multivariable moderator analysis showed a modest negative association with InRR. These results suggest that longer MP exposure may be associated with greater reductions in biomass and NUE, although the magnitude of the linear effect was relatively small. Exposure duration showed little association with the root-to-shoot ratio, whereas longer exposure tended to be associated with lower biomass and NUE, particularly NUE. This pattern suggests that prolonged exposure may gradually affect physiological efficiency before visible growth reductions become apparent [109]. Nitrogen metabolism involves numerous enzyme-mediated processes that may respond more rapidly to chronic environmental stress than structural growth parameters. Particle size showed a contrasting pattern to dose, with positive adjusted coefficients for biomass and NUE. This contrast suggests that larger particles may be less harmful to plant productivity but may still influence root architectural development [110]. Biomass production and nutrient utilization therefore appear more vulnerable to fine and nanoscale particles than to coarse MP fractions. Polymer type also significantly moderated biomass and NUE responses. Among the polymer categories, PVC showed the strongest negative adjusted coefficients relative to PE, particularly for biomass and NUE, indicating that plant responses to MPs may depend substantially on polymer composition. The pronounced negative response associated with PVC may therefore reflect polymer-specific effects rather than particle exposure alone.

## 5. Conclusions

This meta-analysis provides comprehensive evidence that MPs/NPs generally reduce plant biomass and NUE, while effects on root-to-shoot ratio are comparatively weaker and more variable. PVC exhibited the strongest negative effect on biomass and NUE and small MP size showed the strongest adverse effects on biomass and NUE. The study also showed that MPs/NPs can modify soil pH, which plays a central role in regulating nutrient availability, root functioning and microbial activities. Acidic growth medium exposed to MPs exhibited the strongest reductions in biomass and NUE. This work advances the current understanding of how MPs/NPs influence agricultural ecosystems by integrating plant physiological responses with soil environmental processes. However, for further research, long term field-based experiments and environmentally realistic MP/NP concentrations should be prioritized, as MPs/NPs represent an emerging and persistent threat to sustainable crop production. Addressing these would be critical for protecting soil fertility, ensuring long term agricultural sustainability and maintaining crop productivity in an increasing plastic contaminated environment. Limitations of this work include the manner of the experiments, which were short term and undertaken in the lab/greenhouse. It is therefore recommended that more on-field and long-term work on the effects of MPs/NPs on plant germination, growth and metabolism and on soil ecosystems be carried out and reported.

## Figures and Tables

**Figure 1:**
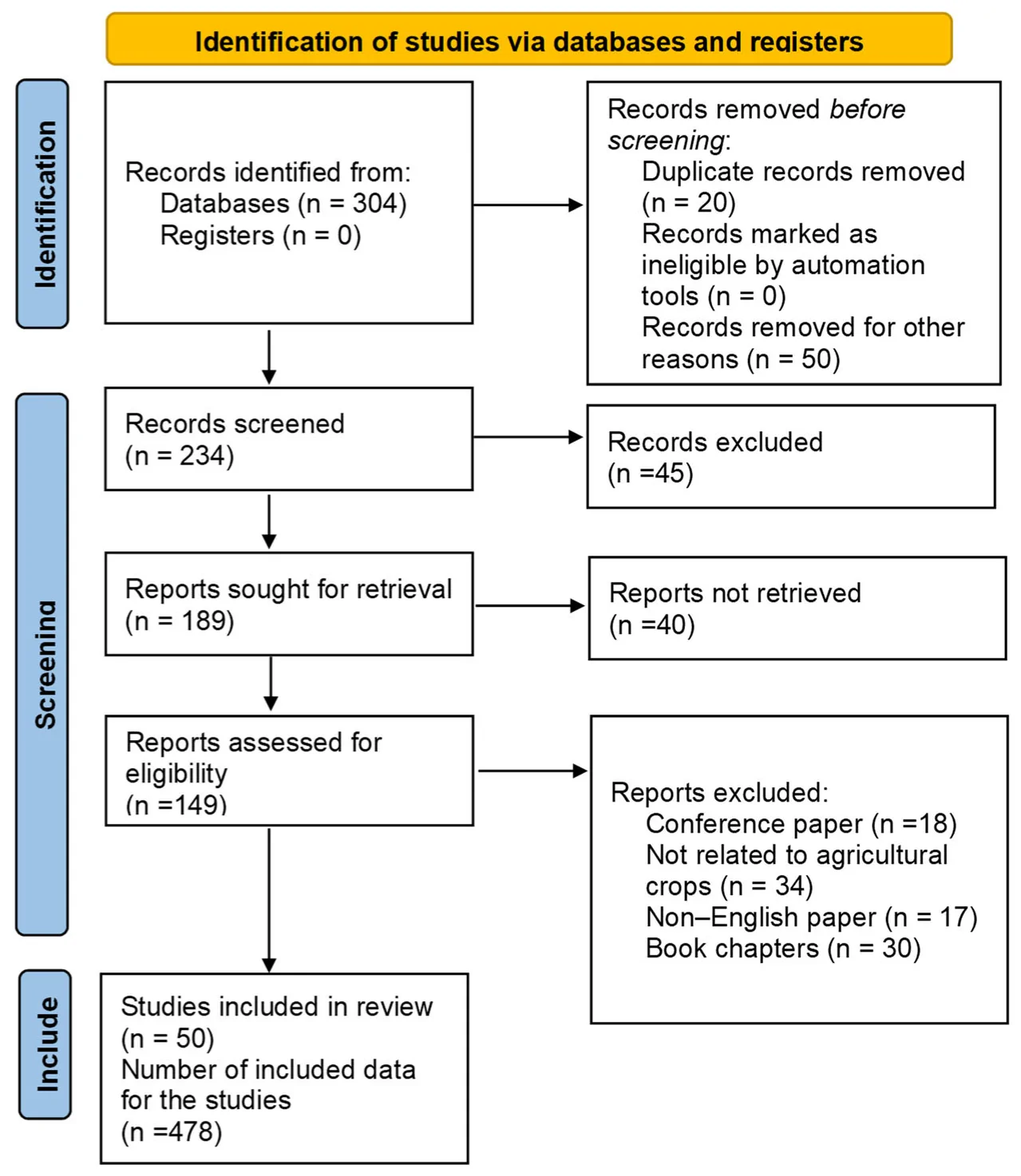
Flow diagram showing the results of the literature search adapted from [20].

**Figure 2:**
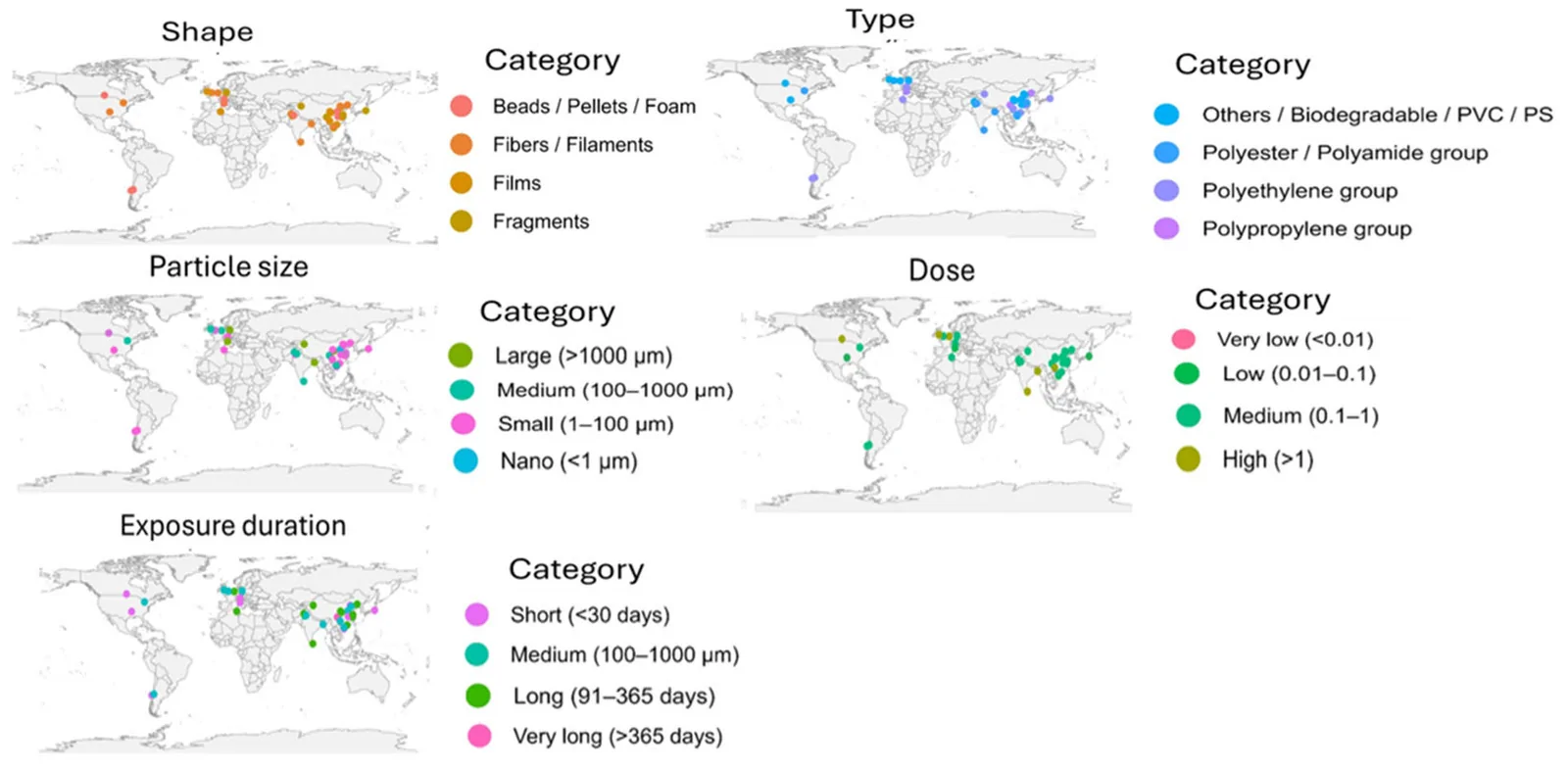
Global distribution of studies on MPs and their characteristics.

**Figure 3:**
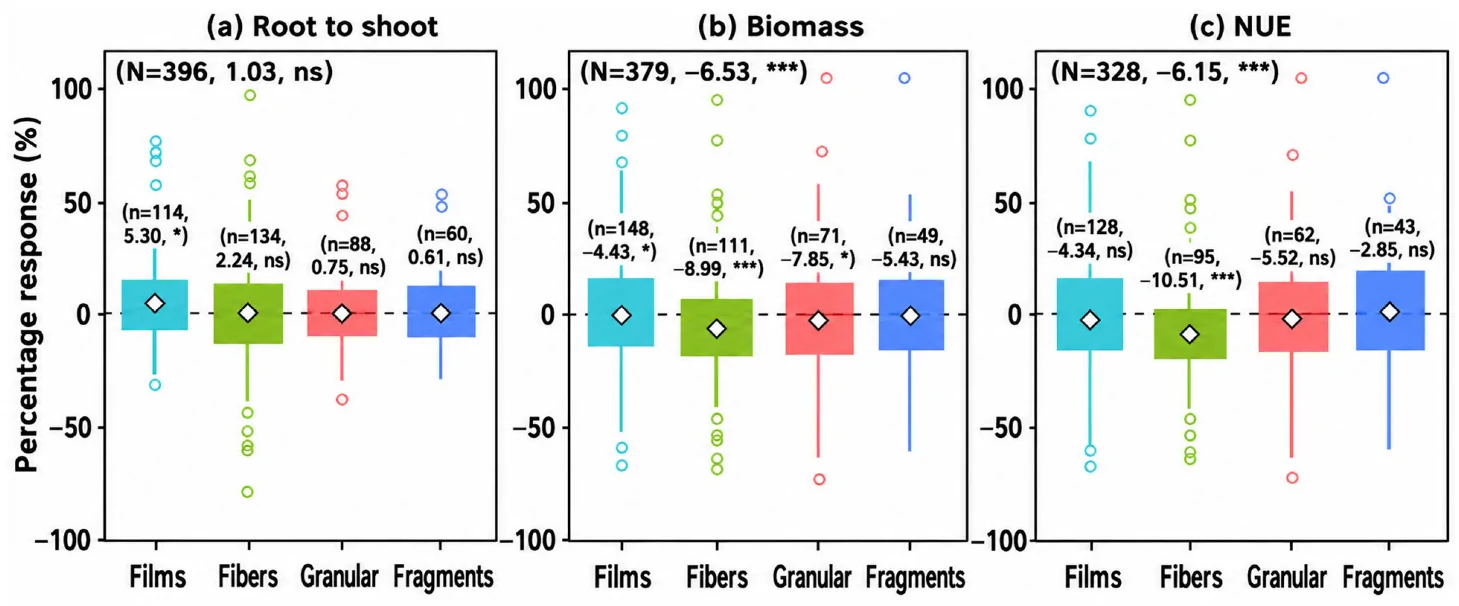
Effects of different MP shapes (films, fibers, granular particles, and fragments) on (**a**) root-to-shoot ratio, (**b**) biomass, and (**c**) nitrogen use efficiency (NUE). Boxes represent the interquartile range (25th–75th percentile), horizontal lines indicate medians, diamonds represent mean percentage responses, whiskers indicate the range of non-outlier observations, and circles represent outliers. The dashed horizontal line indicates no effect relative to control. Asterisks indicate statistical significance (* *p* < 0.05, *** *p* < 0.001; ns = not significant).

**Figure 4:**
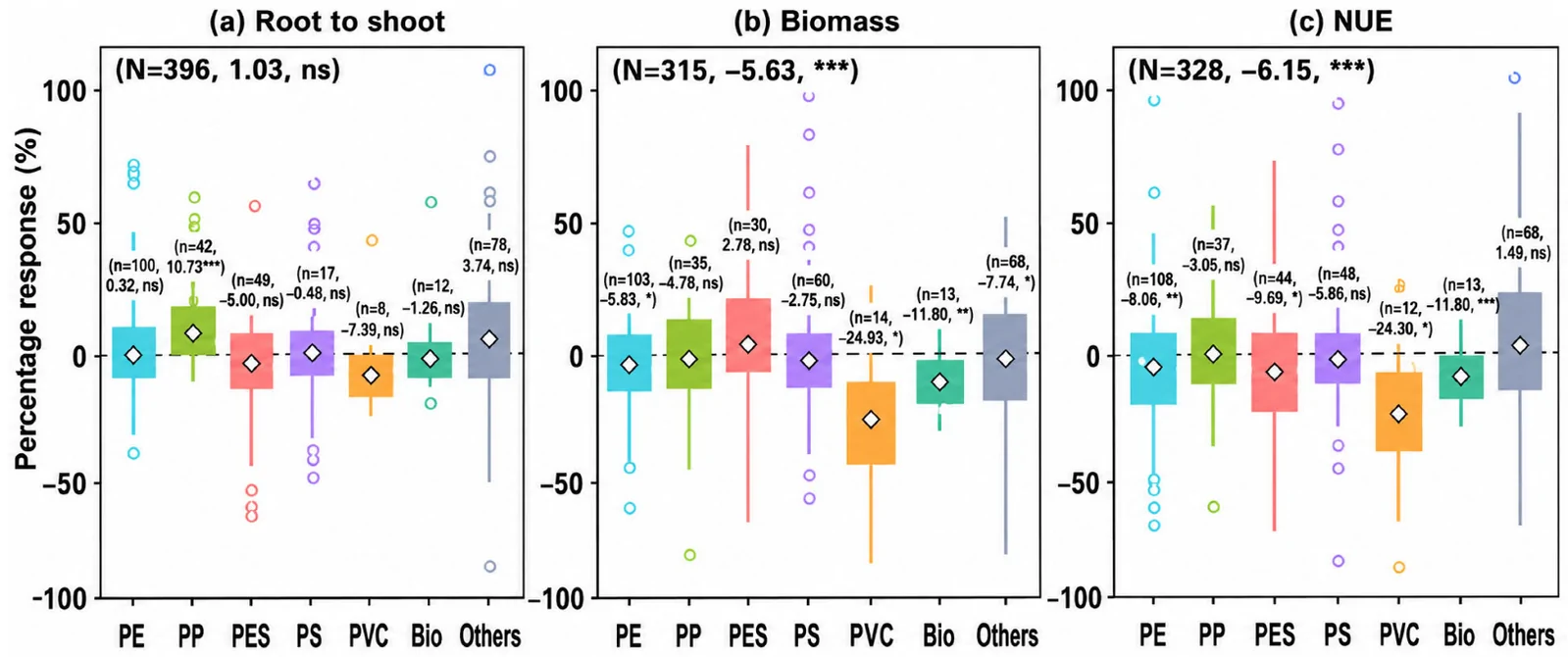
Impact of MP type (polyethylene (PE), polypropylene (PP), poly-ester (PES), polystyrene (PS), polyvinyl chloride (PVC), biodegradable plastics (Bio), and other polymers) on (**a**) root-to-shoot ratio, (**b**) biomass, and (**c**) nitrogen use efficiency (NUE). Boxes rep-resent the interquartile range (25th–75th percentile), horizontal lines indicate medians, diamonds represent mean percentage responses, whiskers indicate the range of non-outlier observations, and circles represent outliers. The dashed horizontal line indicates no effect relative to control. Asterisks indicate statistical significance (* *p* < 0.05, ** *p* < 0.01, *** *p* < 0.001; ns = not significant).

**Figure 5:**
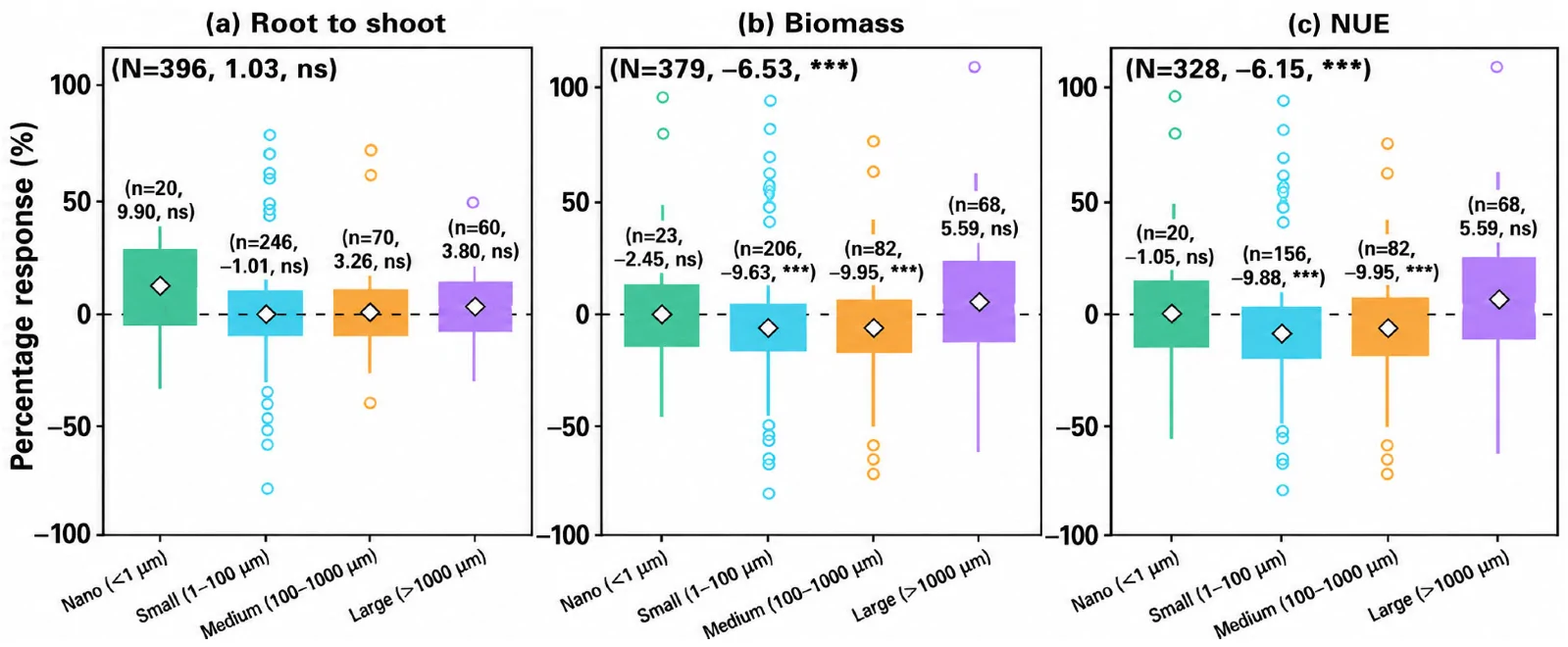
Impact of MP particle size on (**a**) root-to-shoot ratio, (**b**) biomass, and (**c**) nitrogen use efficiency (NUE) of crops. Particle size categories included nano-sized particles (<1 μm), small particles (1–100 μm), medium-sized particles (100–1000 μm), and large particles (>1000 μm). Boxes represent the interquartile range (25th–75th percentile), horizontal lines indicate medians, diamonds represent mean percentage responses, whiskers indicate the range of non-outlier observations, and circles represent outliers. The dashed horizontal line indicates no effect relative to control. Statistical significance is indicated as *** *p* < 0.001, and ns = not significant.

**Figure 6:**
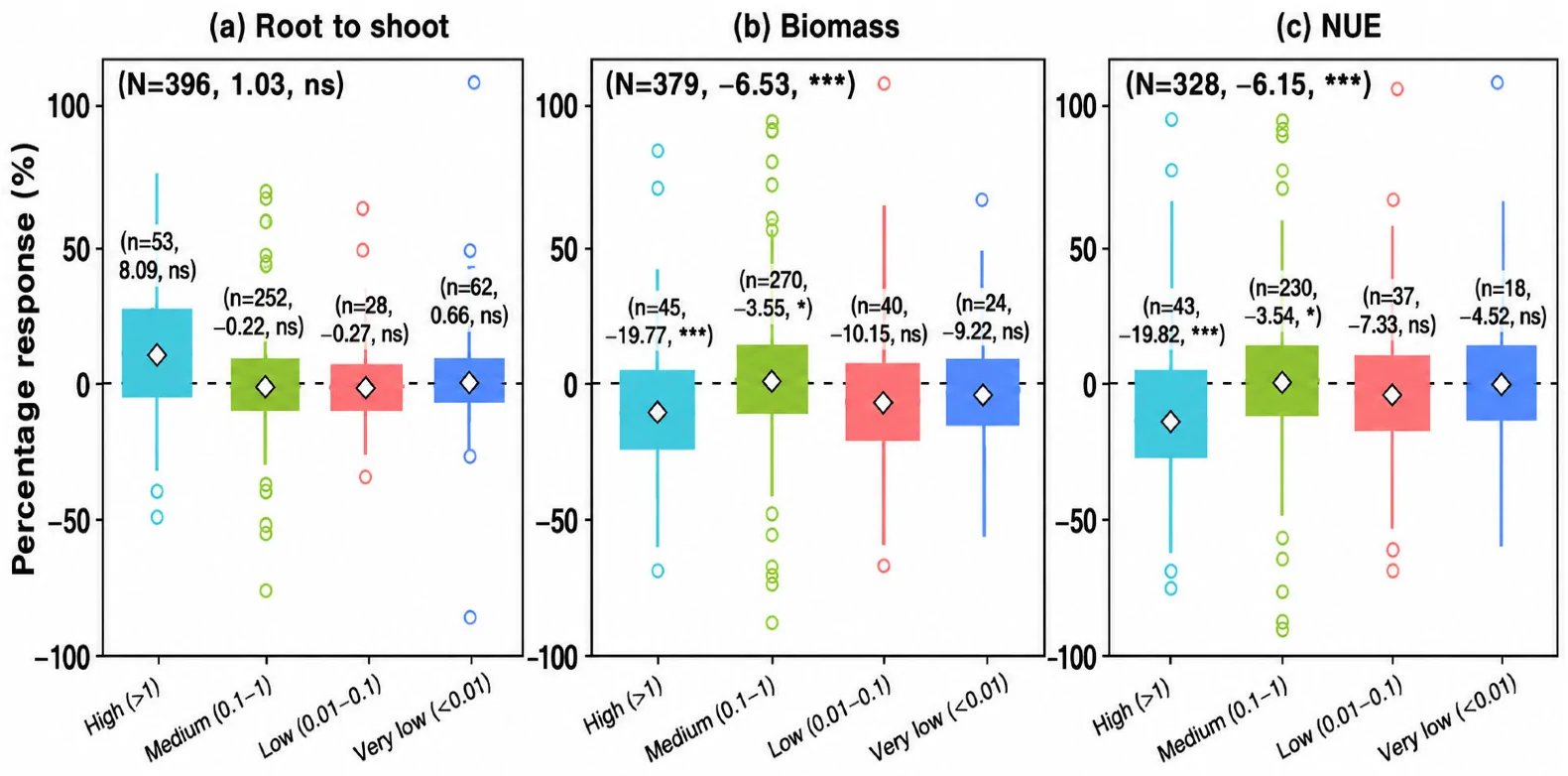
Impact of MP dose on (**a**) root-to-shoot ratio, (**b**) biomass, and (**c**) nitrogen use efficiency (NUE) of crops. Dose categories included high (>1), medium (0.1–1), low (0.01–0.1), and very low (<0.01). Boxes represent the interquartile range (25th–75th percentile), horizontal lines indicate medians, diamonds represent mean percentage responses, whiskers indicate the range of non-outlier observations, and circles represent outliers. The dashed horizontal line denotes no effect relative to the control treatment. Statistical significance is indicated as * *p* < 0.05, *** *p* < 0.001, and ns = not significant.

**Figure 7:**
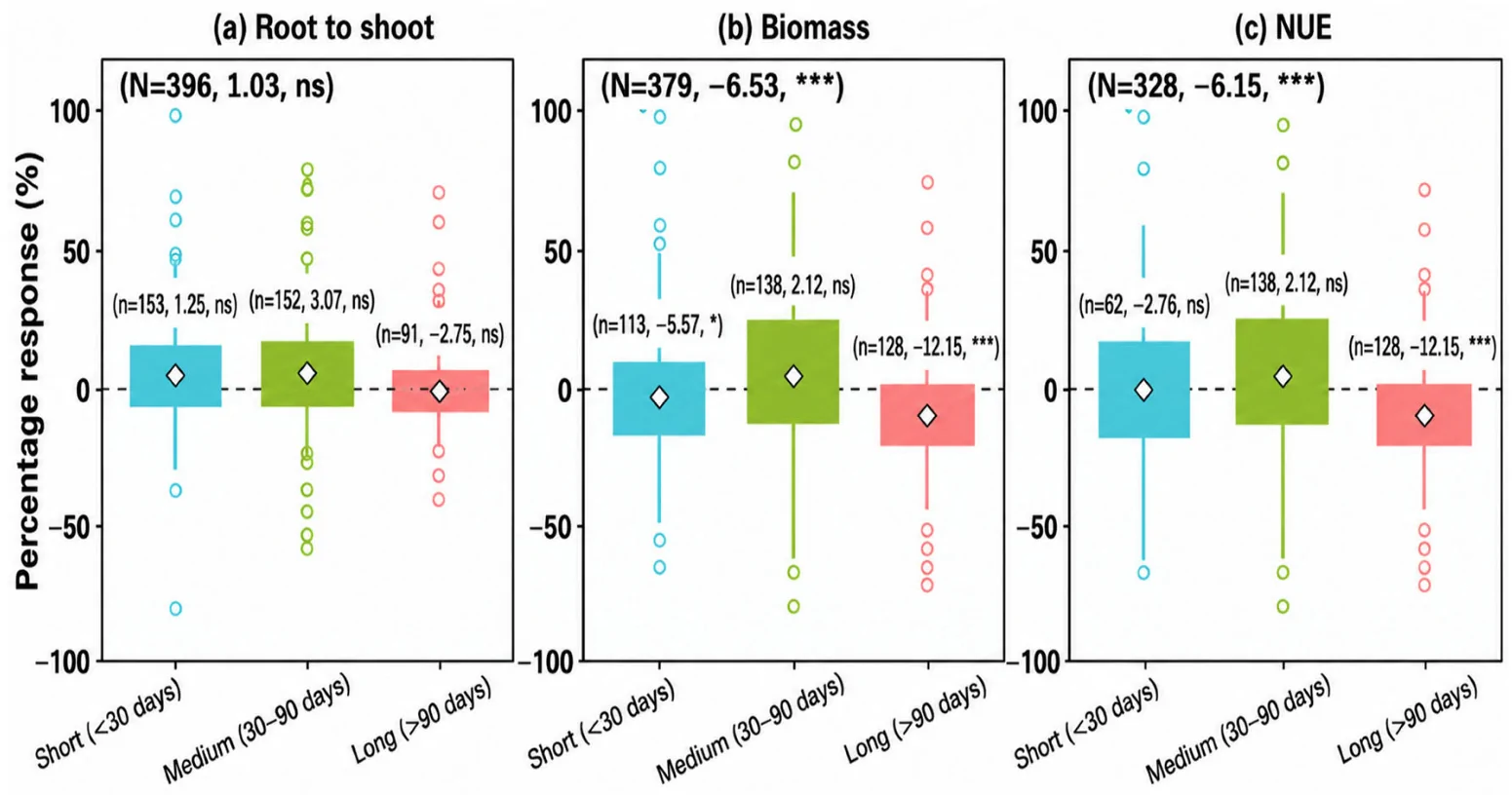
Impact of MP exposure on (**a**) root-to-shoot ratio, (**b**) biomass, and (**c**) nitrogen use efficiency (NUE) of crops. Exposure duration categories included short-term (<30 days), medi-um-term (30–90 days), and long-term (>90 days) treatments. Boxes represent the interquartile range (25th–75th percentile), horizontal lines indicate medians, diamonds represent mean percentage re-sponses, whiskers indicate the range of non-outlier observations, and circles represent outliers. The dashed horizontal line denotes no effect relative to the control treatment. Statistical significance is indicated as * *p* < 0.05, *** *p* < 0.001, and ns = not significant.

**Figure 8:**
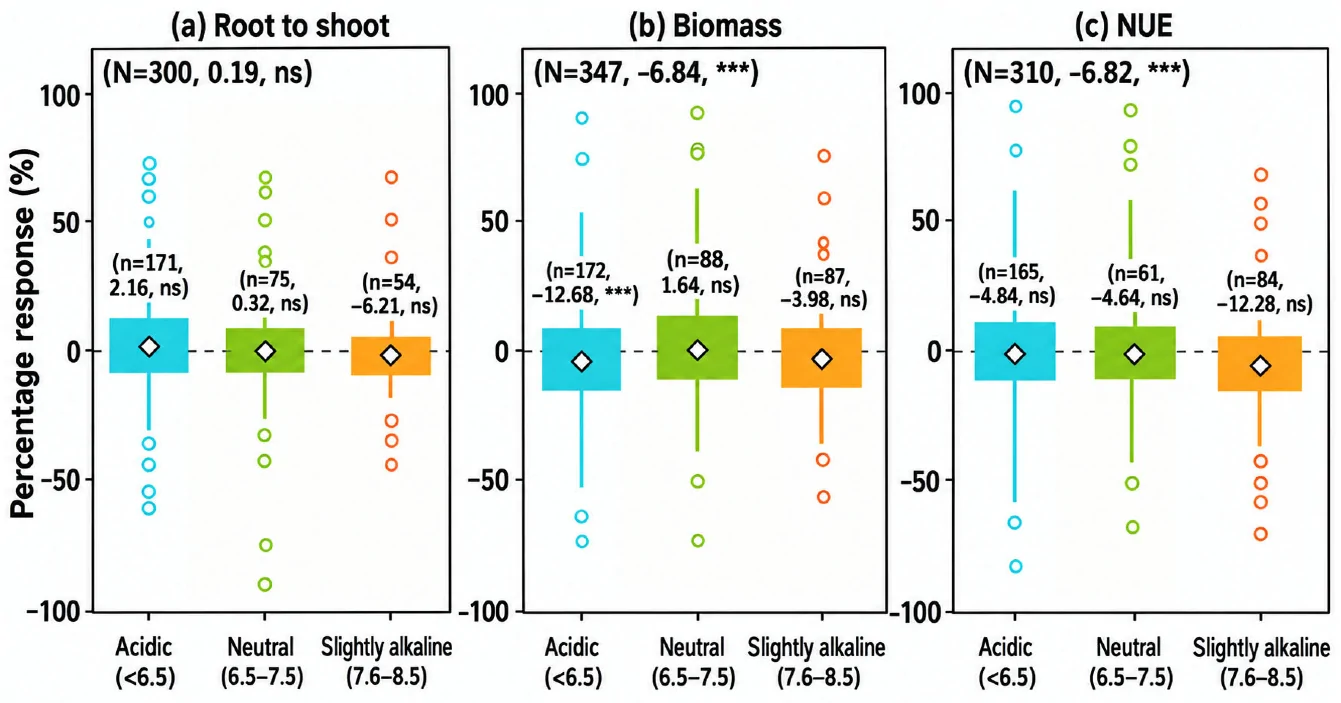
Impact of MPs on the pH of growth medium and their effect on (**a**) root-to-shoot ratio, (**b**) biomass, and (**c**) nitrogen use efficiency (NUE) under MP exposure. Growth media were categorized as acidic (<6.5), neutral (6.5–7.5), and slightly alkaline (7.6–8.5). Boxes represent the in-terquartile range (25th–75th percentile), horizontal lines indicate medians, diamonds represent mean percentage responses, whiskers indicate the range of non-outlier observations, and circles represent outliers. The dashed horizontal line denotes no effect relative to the control treatment. Statistical significance is indicated as *** *p* < 0.001, and ns = not significant.

**Figure 9:**
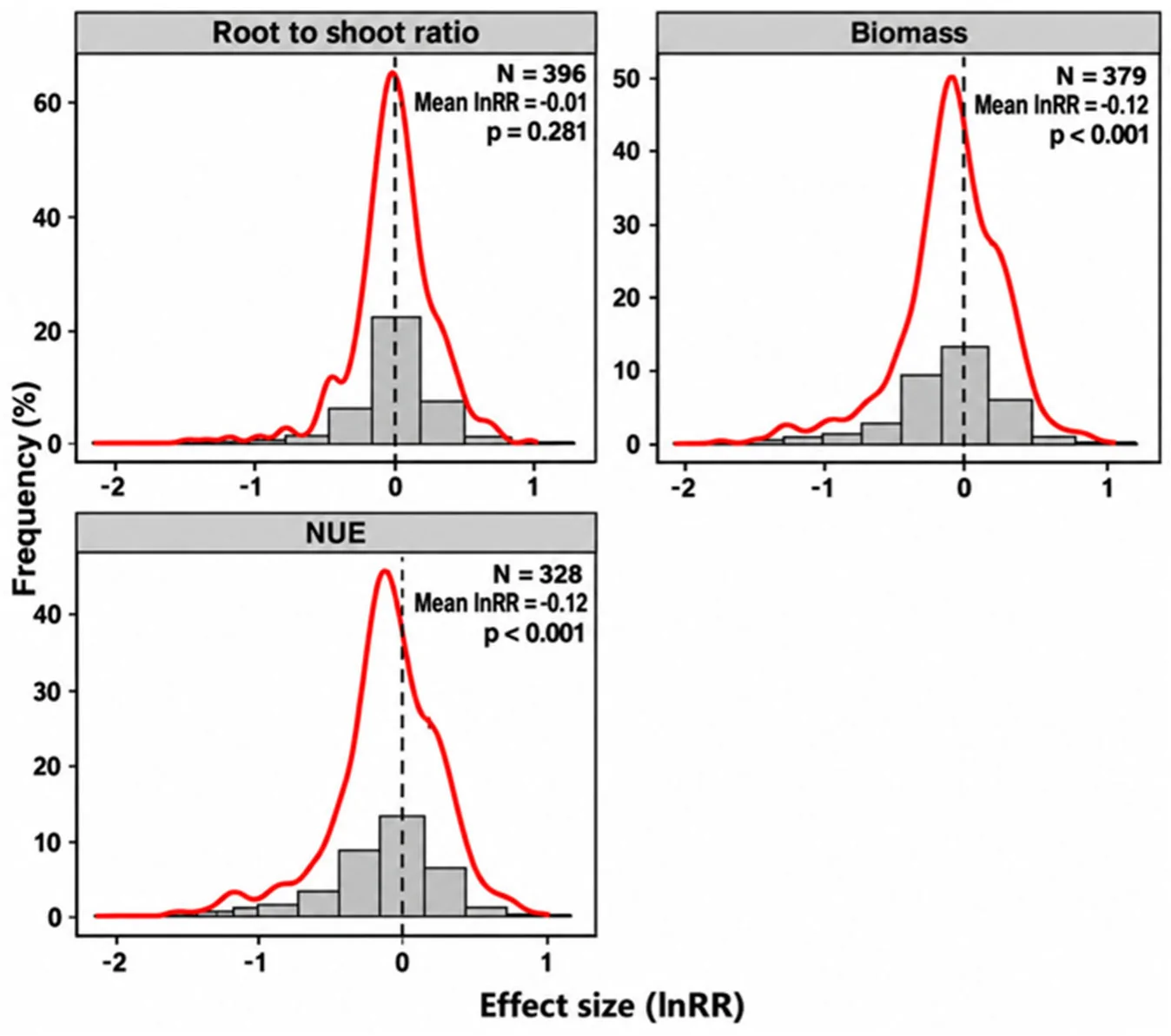
The frequency distributions and density curves of effect sizes for root-to-shoot ratio, biomass and NUE in crops, presented in order to show the overall effects of MPs/NPs. Negative InRR values indicate the inhibitory effects of MPs/NPs on crop performance.

**Figure 10:**
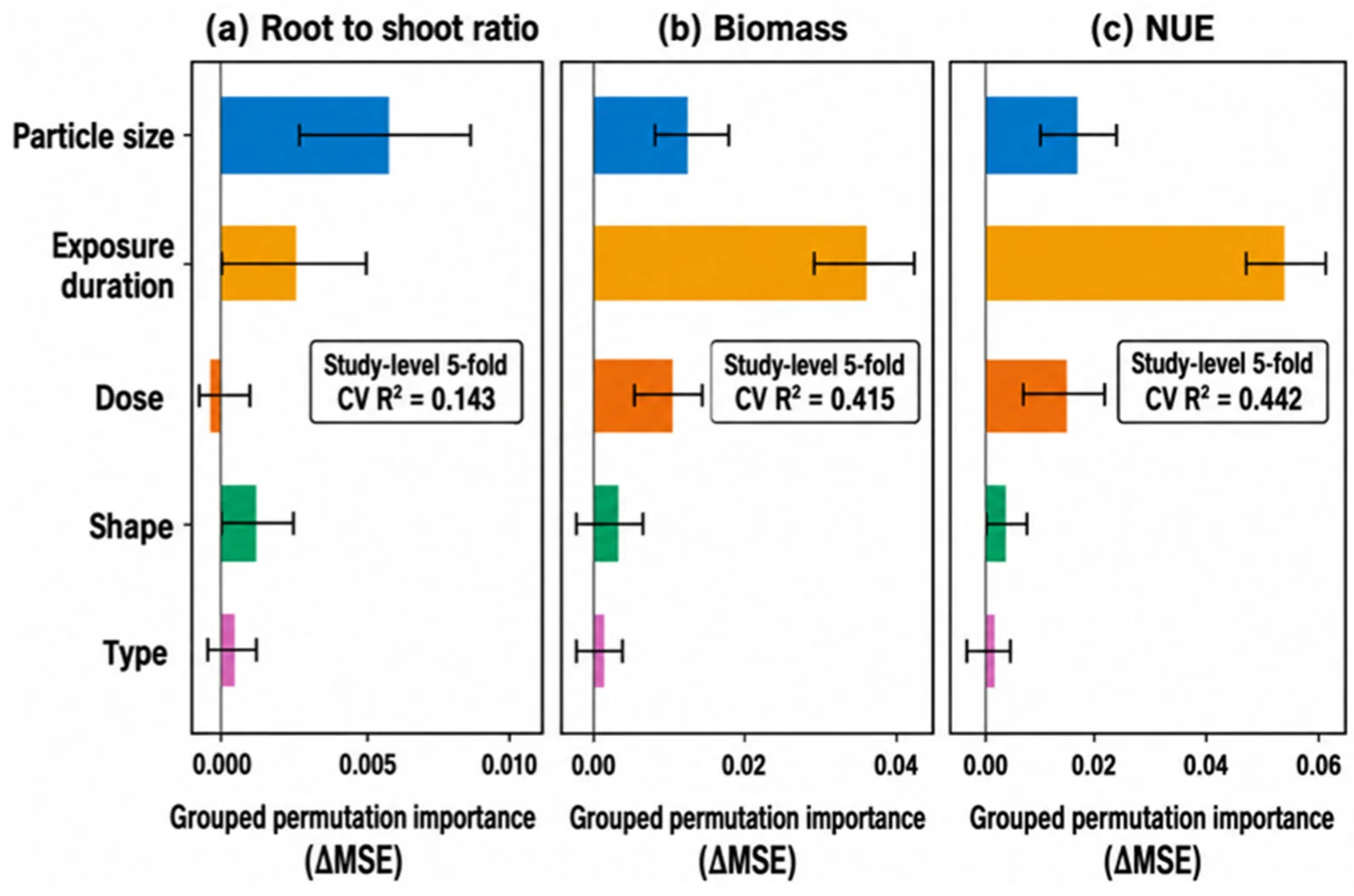
Relative importance of MP characteristics on crop responses. Grouped permutation importance (ΔMSE) of particle size, exposure duration, dose, shape, and polymer type for (**a**) root-to-shoot ratio, (**b**) biomass, and (**c**) nitrogen use efficiency (NUE). Error bars indicate variability in importance estimates, while study-level five-fold CV R2 indicates model predictive performance.

**Figure 11:**
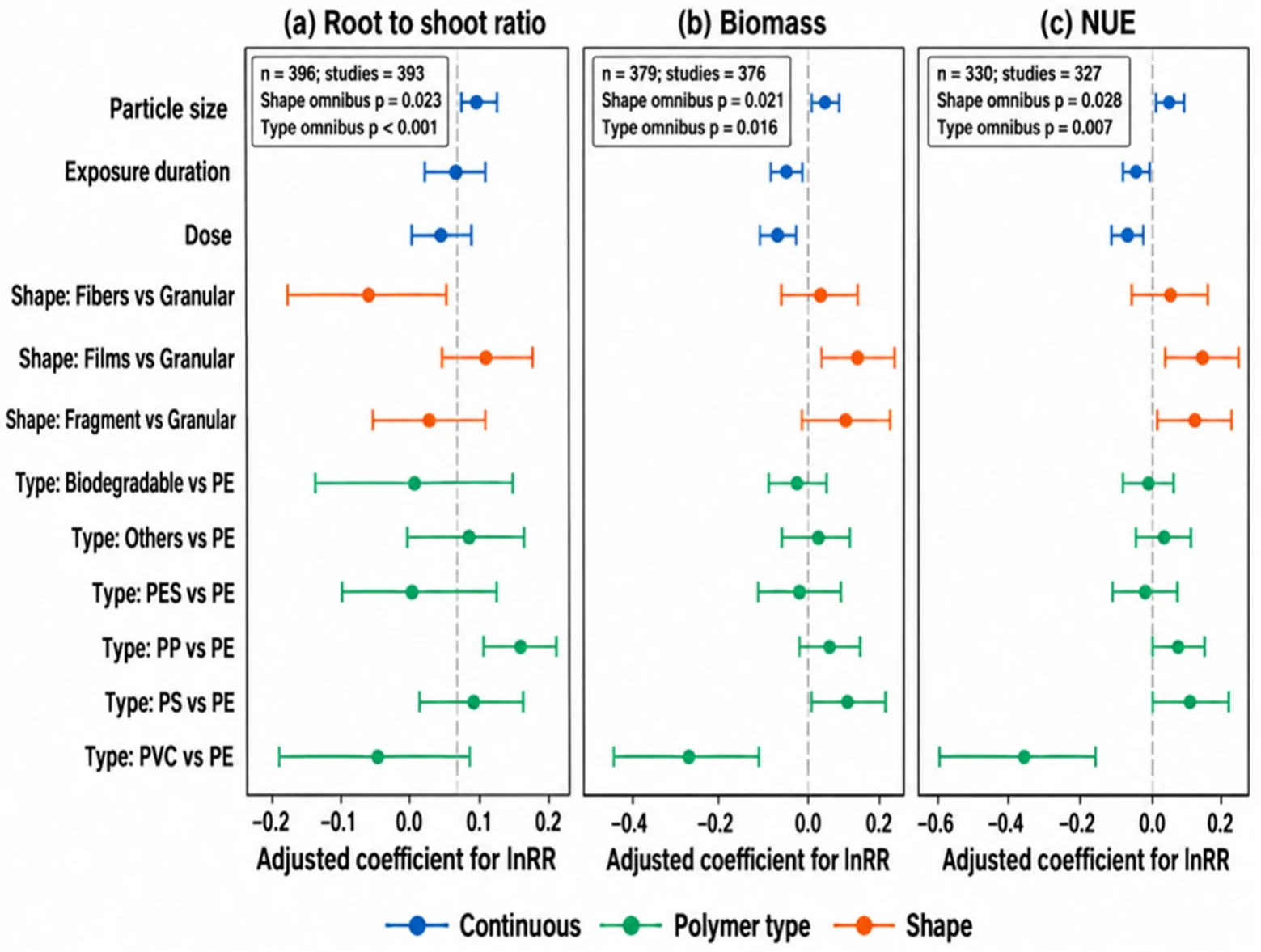
Effects of microplastic characteristics on crop responses based on adjusted regression coefficients. Adjusted regression coefficients and their confidence intervals show the direction and magnitude of the relationships between particle size, dose, exposure duration, shape, and polymer type and the LnRR of (**a**) root-to-shoot ratio, (**b**) biomass, and (**c**) nitrogen use efficiency (NUE). n indicates the number of observations/effect sizes and studies indicates the number of unique studies. The vertical zero line represents no effect, with positive and negative coefficients indicating increases and decreases in the respective crop responses, respectively.

**Table 1:** Classification categories used in microplastic meta-analysis study.

Parameter	Category	Definition/Range
Shape	Films	Film-shaped microplastics
	Fibers	Fiber-shaped microplastics
	Granular	Granular particles
	Fragments	Irregular fragmented particles
Polymer type	PE	Polyethylene
	PP	Polypropylene
	PES	Polyester
	PS	Polystyrene
	PVC	Polyvinyl chloride
	Bio	Biodegradable plastics
	Others	Other polymer types
Particle size	Nano	<1 µm
	Small	1–100 µm
	Medium	100–1000 µm
	Large	>1000 µm
Dose	Very Low	<0.01
	Low	0.01–0.1
	Medium	0.1–1
	High	>1
Exposure duration	Short	<30 days
	Medium	30–90 days
	Long	>90 days
Growth medium pH	Acidic	<6.5
	Neutral	6.5–7.5
	Slightly alkaline	7.6–8.5

## Data Availability

No new data were created or analyzed in this studies, further inquiries about the data supporting this study should be made to the corresponding author.

## Supplementary Materials

The following supporting information can be downloaded at https://www.mdpi.com/article/10.3390/toxics14100860/s1, Supplementary file: PRISMA checklist; References [10,16,47,92,102,111,112,113,114,115,116,117,118,119,120,121,122,123,124,125,126,127,128,129,130,131,132,133,134,135,136,137,138,139,140,141,142,143,144,145,146,147,148,149,150,151,152,153,154,155] for the studies used in the meta-analysis are cited in the Supplementary Materials. Table S1: Literature search strategy used for study identification; Table S2: Summary of the inclusion and exclusion criteria used for study selection; Table S3: Heterogeneity statistics for the effects of MP/nanoplastic on root, shoot, biomass and NUE across MP shapes; Table S4: Heterogeneity statistics for the effects of MP/nanoplastic on root, shoot, biomass and NUE across MP types; Table S5: Heterogeneity statistics for the effects of MP/nanoplastic on root, shoot, biomass and NUE across MP particle size; Table S6: Heterogeneity statistics for the effects of MP/nanoplastic on root, shoot, biomass and NUE across MP dose; Table S7: Heterogeneity statistics for the effects of MP/nanoplastic on root, shoot, biomass and NUE across MP exposure duration; Table S8: Heterogeneity statistics for the effects of MP/nanoplastic on root, shoot, biomass and NUE across MP growth medium; Table S9: Overall heterogeneity statistics for the effects of MP/nanoplastic on root, shoot, biomass and NUE based on Cochran’s Q test and I^2^ statistics; Figure S1. Funnel plot of effect size (InRR) of author publication bias before and after trim and fill.

## Abbreviations

The following abbreviations are used in this manuscript:

BPs	Biodegradable plastics
MPs	Microplastics
NPs	Nanoplastics
PE	Polyethylene
PET	Polyethylene terephthalate
PLA	Polylactic acid
PP	Polypropylene
PS	Polystyrene
PVC	Polyvinyl chloride
ROS	Reactive oxygen species
