# Supplementary material for: Effects of Micro/Nanoplastics on Crop Physiological Responses and Soil Environment: A Meta-Analysis
Source: Toxics. 2026 Sep 28;14(10):860. doi: 10.3390/toxics14100860 (PMC13709670; doi:10.3390/toxics14100860)
Supplement: Supplementary file 1 [file toxics-14-00860-s001.zip › toxics-4558389-Supplementary file.pdf]

toxics

MDPI

*Systematic Review*

# Effects of Micro/Nano-Plastics on Crop Physiological Responses and Soil Environment: A Meta-Analysis

**Aaron Ohene Boanor [1], Rose Nimoh Serwaa [1], Jin Hee Park [2,*] and Jwakyung Sung [1,*]**

[1] Department of Crop Science, College of Agriculture, Life and Environment Science, Chungbuk National University, Cheongju-si 28666, Chungcheongbuk-do, Republic of Korea; aaronboanor05@chungbuk.ac.kr (A.O.B.); roseserwaanimoh47@chungbuk.ac.kr (R.N.S.)
[2] Department of Environmental and Biological Chemistry, College of Agriculture, Life and Environment Science, Chungbuk National University, Cheongju-si 28666, Chungcheongbuk-do, Republic of Korea
* Correspondence: pjinh@chungbuk.ac.kr (J.H.P.); jksung73@chungbuk.ac.kr (J.S.)

## Supplementary Tables and Figure

- Table S1. Literature search strategy used for study identification
- Table S2. Summary of the inclusion and exclusion criteria used for study selection
- **Table S3.** Heterogeneity statistics for the effects of MP/ Nano-plastic on root: shoot, biomass and NUE across MP shapes
- **Table S4.** Heterogeneity statistics for the effects of MP/ Nano-plastic on root: shoot, biomass and NUE across MP types
- **Table S5.** Heterogeneity statistics for the effects of MP/ Nano-plastic on root: shoot, biomass and NUE across MP particle size
- **Table S6.** Heterogeneity statistics for the effects of MP/ Nano-plastic on root: shoot, biomass and NUE across MP dose
- **Table S7.** Heterogeneity statistics for the effects of MP/ Nano-plastic on root: shoot, biomass and NUE across MP exposure duration
- **Table S8.** Heterogeneity statistics for the effects of MP/ Nano-plastic on root: shoot, biomass and NUE across MP growth medium
- **Table S9.** Overall heterogeneity statistics for the effects of MP/ Nano-plastic on root: shoot, biomass and NUE based on Cochran's Q test and $I^2$ statistics
- **Figure S1**. Funnel plot of effect size (InRR) of author publication bias before and after trim and fill

## A. Supplementary tables

Table S1. Literature search strategy used for study identification

| Database | Search strategy | Publication Years | Language | Search Date | Records Retrieved |
|---|---|---|---|---|---|
| Web of Science Core Collection | TS=("microplastics" OR "nanoplastics") AND (crop* OR plant* OR agriculture*) AND (biomass OR "root to shoot ratio" OR "nutrient use efficiency" OR NUE OR "plant growth" OR "soil pH") | 2013–2025 | English | 2025 | 180 |
| Scopus | ("microplastics" OR "nanoplastics") AND (crop* OR plant* OR agriculture*) AND (biomass OR "root to shoot ratio" OR "nutrient use efficiency" OR NUE OR "plant growth" OR "soil pH") | 2013–2025 | English | 2025 | 50 |
| Google Scholar | Keywords searched: microplastics AND crops; microplastics AND biomass; microplastics AND nutrient use efficiency; nanoplastics AND plant growth; microplastics AND root to shoot ratio; microplastics AND soil pH; microplastics AND agricultural systems. | 2013–2025 | English | 2025 | 66 |
| Reference lists | Reference lists of relevant review articles and published studies were manually screened to identify additional eligible studies not captured during the electronic database search. | 2013–2025 | English | Same as above | 8 |

Table S2. Summary of the inclusion and exclusion criteria used for study selection

| **Criterion** | **Inclusion Criteria** | **Exclusion Criteria** |
|---|---|---|
| Language | Articles published in English. | Articles not published in English. |
| Publication type | Original peer-reviewed research articles. | Review articles, conference papers, book chapters, non-peer-reviewed reports, and other grey literature. |
| Study focus | Studies evaluating the effects of microplastics (MPs) or nanoplastics (NPs) on crop biomass, root to shoot ratio, and/or nutrient use efficiency (NUE). | Studies unrelated to agricultural crops, aquatic ecosystem studies, or studies not reporting the target response variables. |
| Experimental system | Soil-based crop production systems compare control treatment with MP or NP exposure. | Studies lacking an appropriate control comparison or conducted solely as modeling or simulation studies. |
| Response variables | Reported at least one target response variable (biomass, root to shoot ratio, or NUE). | Studies that did not report any of the target response variables. |
| Data requirements | Provided sufficient statistical information for effect size calculation (mean, SD or SE, and sample size). | Studies with insufficient quantitative data for meta-analysis. |
| Duplicate records | Most complete or latest version retained. | Duplicate publications or overlapping datasets were removed before screening. |

Variables extracted from each eligible study

- Crop variables: crop species, biomass, root to shoot ratio, and nutrient use efficiency (NUE).
- Microplastic variables: polymer type (PE, PP, PVC, PS, PET, biodegradable plastics), particle size (nano, small, medium, large), shape (films, fibers, fragments, granular), concentration (dose), and exposure duration.
- Environmental variables: soil pH (growth medium pH) and experimental conditions.

**Table S3.** Heterogeneity statistics for the effects of MP/ Nano-plastic on root: shoot, biomass and NUE across MP shapes

| Response | Shape Factor | Subgroup | k | Q | $I^2$_percent | DF | $Tau^2$ | Q_pvalue |
|---|---|---|---|---|---|---|---|---|
| Root:shoot | MP shape | Overall | 396 | 1324.8 | 73.17 | 395 | 0.0392 | <0.001 |
| Root:shoot | MP shape | Films | 114 | 273.64 | 58.89 | 113 | 0.0205 | <0.001 |
| Root:shoot | MP shape | Fibers | 134 | 689.89 | 84.48 | 133 | 0.0949 | <0.001 |
| Root:shoot | MP shape | Granular | 88 | 241.88 | 63.92 | 87 | 0.0185 | <0.001 |
| Root:shoot | MP shape | Fragments | 60 | 78.43 | 21.14 | 59 | 0.0048 | 0.046 |
| Biomass | MP shape | Overall | 379 | 3159.64 | 88.5 | 378 | 0.0923 | <0.001 |
| Biomass | MP shape | Films | 148 | 1297.82 | 89.76 | 147 | 0.0839 | <0.001 |
| Biomass | MP shape | Fibers | 111 | 572.82 | 80.13 | 110 | 0.0753 | <0.001 |
| Biomass | MP shape | Granular | 71 | 860.19 | 92.9 | 70 | 0.1343 | <0.001 |
| Biomass | MP shape | Fragments | 49 | 380.19 | 86.2 | 48 | 0.0961 | <0.001 |
| NUE | MP shape | Overall | 328 | 3025.24 | 89.77 | 327 | 0.1017 | <0.001 |
| NUE | MP shape | Films | 128 | 1265.51 | 91.26 | 127 | 0.0959 | <0.001 |
| NUE | MP shape | Fibers | 95 | 546.19 | 82.55 | 94 | 0.0841 | <0.001 |
| NUE | MP shape | Granular | 62 | 820.4 | 93.47 | 61 | 0.1406 | <0.001 |
| NUE | MP shape | Fragments | 43 | 339.83 | 85.74 | 42 | 0.1001 | <0.001 |

Where; MP = Micro plastics; Q = Cochran's heterogeneity statistic; $I^2$ = percentage of total variation attributable to heterogeneity; DF = degrees of freedom; $Tau^2$ = estimated between–study variance

**Table S4.** Heterogeneity statistics for the effects of MP/ Nano-plastic on root: shoot, biomass and NUE across MP types

| Response | Type Factor | Subgroup | k | Q | $I^2$ | DF | $Tau^2$ | Q_pvalue |
|---|---|---|---|---|---|---|---|---|
| Root:shoot | MP type | Overall | 396 | 1324.8 | 73.17 | 395 | 0.0392 | <0.001 |
| Root:shoot | MP type | PE | 100 | 259.18 | 62.38 | 99 | 0.0223 | <0.001 |
| Root:shoot | MP type | PP | 42 | 29.63 | 0 | 41 | 0 | 0.906 |
| Root:shoot | MP type | PES | 49 | 280.16 | 85.33 | 48 | 0.0965 | <0.001 |
| Root:shoot | MP type | PS | 107 | 218.04 | 51.24 | 106 | 0.0174 | <0.001 |
| Root:shoot | MP type | PVC | 8 | 9.64 | 27.4 | 7 | 0.0101 | 0.21 |
| Root:shoot | MP type | Bio | 12 | 16.67 | 38.24 | 11 | 0.0128 | 0.118 |
| Root:shoot | MP type | Others | 78 | 459.19 | 89.37 | 77 | 0.0912 | <0.001 |
| Biomass | MP type | Overall | 315 | 1703.84 | 81.07 | 314 | 0.0524 | <0.001 |
| Biomass | MP type | PE | 103 | 441.95 | 73.59 | 102 | 0.0244 | <0.001 |
| Biomass | MP type | PP | 35 | 134.59 | 76.33 | 34 | 0.0551 | <0.001 |
| Biomass | MP type | PES | 30 | 124.5 | 77.88 | 29 | 0.059 | <0.001 |
| Biomass | MP type | PS | 60 | 258.19 | 71.72 | 59 | 0.0399 | <0.001 |
| Biomass | MP type | PVC | 11 | 105.91 | 87.79 | 10 | 0.1351 | <0.001 |
| Biomass | MP type | Bio | 13 | NA | NA | 12 | NA | NA |
| Biomass | MP type | Others | 63 | 493.3 | 88.3 | 62 | 0.096 | <0.001 |
| NUE | MP type | Overall | 328 | 3025.24 | 89.77 | 327 | 0.1017 | <0.001 |
| NUE | MP type | PE | 106 | 1055.75 | 91.81 | 105 | 0.0996 | <0.001 |
| NUE | MP type | PP | 37 | 148.36 | 77.07 | 36 | 0.0573 | <0.001 |
| NUE | MP type | PES | 44 | 491.25 | 90.28 | 43 | 0.1492 | <0.001 |
| NUE | MP type | PS | 48 | 399.78 | 86.92 | 47 | 0.0953 | <0.001 |
| NUE | MP type | PVC | 12 | 106.94 | 85.41 | 11 | 0.1342 | <0.001 |
| NUE | MP type | Bio | 13 | NA | NA | 12 | NA | NA |
| NUE | MP type | Others | 68 | 703.24 | 91.38 | 67 | 0.1058 | <0.001 |

Where; MP = Micro plastics; Q = Cochran's heterogeneity statistic; $I^2$ = percentage of total variation attributable to heterogeneity; DF = degrees of freedom; $Tau^2$ = estimated between–study variance

**Table S5.** Heterogeneity statistics for the effects of MP/ Nano-plastic on root: shoot, biomass and NUE across MP particle size

| Response | Particle Size Factor | Subgroup | k | Q | $I^2$ | DF | $Tau^2$ | Q_pvalue |
|---|---|---|---|---|---|---|---|---|
| Root:shoot | Particle size | Overall | 396 | 1324.8 | 73.18 | 395 | 0.0392 | <0.001 |
| Root:shoot | Particle size | Nano (<1 µm) | 20 | 82.42 | 82.69 | 19 | 0.0397 | <0.001 |
| Root:shoot | Particle size | Small (1–100 µm) | 246 | 909.96 | 76.22 | 245 | 0.0531 | <0.001 |
| Root:shoot | Particle size | Medium (100–1000 µm) | 70 | 228.24 | 70.64 | 69 | 0.0269 | <0.001 |
| Root:shoot | Particle size | Large (>1000 µm) | 60 | 65.74 | 0 | 59 | 0 | 0.255 |
| Biomass | Particle size | Overall | 379 | 3159.64 | 88.5 | 378 | 0.0923 | <0.001 |
| Biomass | Particle size | Nano (<1 µm) | 23 | 272.15 | 90.6 | 22 | 0.0882 | <0.001 |
| Biomass | Particle size | Small (1–100 µm) | 206 | 1654.47 | 88.09 | 205 | 0.0845 | <0.001 |
| Biomass | Particle size | Medium (100–1000 µm) | 82 | 746.11 | 89.46 | 81 | 0.1038 | <0.001 |
| Biomass | Particle size | Large (>1000 µm) | 68 | 318.33 | 82.96 | 67 | 0.079 | <0.001 |
| NUE | Particle size | Overall | 328 | 3025.24 | 89.77 | 327 | 0.1017 | <0.001 |
| NUE | Particle size | Nano (<1 µm) | 20 | 271.14 | 92.41 | 19 | 0.1012 | <0.001 |
| NUE | Particle size | Small (1–100 µm) | 158 | 1522.67 | 90.35 | 157 | 0.0997 | <0.001 |
| NUE | Particle size | Medium (100–1000 µm) | 82 | 746.11 | 89.46 | 81 | 0.1038 | <0.001 |
| NUE | Particle size | Large (>1000 µm) | 68 | 318.33 | 82.96 | 67 | 0.079 | <0.001 |

Where; MP = Micro plastics; Q = Cochran's heterogeneity statistic; $I^2$ = percentage of total variation attributable to heterogeneity; DF = degrees of freedom; $Tau^2$ = estimated between–study variance

**Table S6.** Heterogeneity statistics for the effects of MP/Nano-plastic on root: shoot, biomass and NUE across MP dose

| Response | Dose Factor | Subgroup | k | Q | $I^2$ | DF | $Tau^2$ | Q_pvalue |
|---|---|---|---|---|---|---|---|---|
| Root:shoot | Dose | Overall | 396 | 1324.8 | 73.18 | 395 | 0.0392 | <0.001 |
| Root:shoot | Dose | High (>1) | 53 | 323.42 | 90.38 | 52 | 0.1022 | <0.001 |
| Root:shoot | Dose | Medium (0.1–1) | 252 | 607.14 | 59.39 | 251 | 0.0215 | <0.001 |
| Root:shoot | Dose | Low (0.01–.1) | 29 | 50.63 | 44.88 | 28 | 0.0139 | 0.006 |
| Root:shoot | Dose | Very low (<0.01) | 62 | 301.98 | 84.03 | 61 | 0.0874 | <0.001 |
| Biomass | Dose | Overall | 379 | 3159.64 | 88.5 | 378 | 0.0923 | <0.001 |
| Biomass | Dose | High (>1) | 45 | 689.73 | 95.08 | 44 | 0.1828 | <0.001 |
| Biomass | Dose | Medium (0.1–1) | 270 | 1595.91 | 82.87 | 269 | 0.0635 | <0.001 |
| Biomass | Dose | Low (0.01–.1) | 40 | 472.49 | 94.06 | 39 | 0.1334 | <0.001 |
| Biomass | Dose | Very low (<0.01) | 24 | 123.14 | 83.63 | 23 | 0.0928 | <0.001 |
| NUE | Dose | Overall | 328 | 3025.24 | 89.77 | 327 | 0.1017 | <0.001 |
| NUE | Dose | High (>1) | 43 | 687.14 | 95.26 | 42 | 0.1921 | <0.001 |
| NUE | Dose | Medium (0.1–1) | 230 | 1545.79 | 85.15 | 229 | 0.0734 | <0.001 |
| NUE | Dose | Low (0.01–.1) | 37 | 419.74 | 93.76 | 36 | 0.1208 | <0.001 |
| NUE | Dose | Very low (<0.01) | 18 | 102 | 86.06 | 17 | 0.109 | <0.001 |

Where; MP = Micro plastics; Q = Cochran's heterogeneity statistic; $I^2$ = percentage of total variation attributable to heterogeneity; DF = degrees of freedom; $Tau^2$ = estimated between–study variance

**Table S7.** Heterogeneity statistics for the effects of MP/ Nano-plastic on root: shoot, biomass and NUE across MP exposure duration

| Response | Exposure Duration Factor | Subgroup | k | Q | $I^2$ | DF | $Tau^2$ | Q_pvalue |
|---|---|---|---|---|---|---|---|---|
| Root:shoot | Exposure duration | Overall | 396 | 1324.8 | 73.18 | 395 | 0.0392 | <0.001 |
| Root:shoot | Exposure duration | Short (<30 days) | 153 | 569.34 | 77.5 | 152 | 0.0475 | <0.001 |
| Root:shoot | Exposure duration | Medium (30–90 days) | 152 | 529.54 | 75.03 | 151 | 0.0462 | <0.001 |
| Root:shoot | Exposure duration | Long (>90 days) | 91 | 214.12 | 58.54 | 90 | 0.0198 | <0.001 |
| Biomass | Exposure duration | Overall | 379 | 3159.64 | 88.5 | 378 | 0.0923 | <0.001 |
| Biomass | Exposure duration | Short (<30 days) | 113 | 701.33 | 84.21 | 112 | 0.07 | <0.001 |
| Biomass | Exposure duration | Medium (30–90 days) | 138 | 1197.95 | 88.86 | 137 | 0.1133 | <0.001 |
| Biomass | Exposure duration | Long (>90 days) | 128 | 1234.29 | 89.7 | 127 | 0.0846 | <0.001 |
| NUE | Exposure duration | Overall | 328 | 3025.24 | 89.77 | 327 | 0.1017 | <0.001 |
| NUE | Exposure duration | Short (<30 days) | 62 | 568.53 | 89.97 | 61 | 0.1055 | <0.001 |
| NUE | Exposure duration | Medium (30–90 days) | 138 | 1197.95 | 88.86 | 137 | 0.1133 | <0.001 |
| NUE | Exposure duration | Long (>90 days) | 128 | 1234.29 | 89.7 | 127 | 0.0846 | <0.001 |

Where; MP = Micro plastics; Q = Cochran's heterogeneity statistic; $I^2$ = percentage of total variation attributable to heterogeneity; DF = degrees of freedom; $Tau^2$ = estimated between–study variance

**Table S8.** Heterogeneity statistics for the effects of MP/ Nano-plastic on root: shoot, biomass and NUE across MP growth medium

| Response | Growth medium | Subgroup | k | Q | I2 | DF | Tau2 | Q_pvalue |
|---|---|---|---|---|---|---|---|---|
| Root:shoot | pH | Overall | 300 | 1184.31 | 78.36 | 299 | 0.0523 | <0.001 |
| Root:shoot | pH | Acidic (<6.5) | 171 | 648.11 | 78.08 | 170 | 0.0452 | <0.001 |
| Root:shoot | pH | Neutral (6.5–7.5) | 75 | 417.66 | 84.9 | 74 | 0.1016 | <0.001 |
| Root:shoot | pH | Slightly alkaline (7.6–8.5) | 54 | 83.23 | 35.8 | 53 | 0.0097 | 0.005 |
| Biomass | pH | Overall | 347 | 3005.56 | 88.98 | 346 | 0.0975 | <0.001 |
| Biomass | pH | Acidic (<6.5) | 172 | 1769.34 | 90.79 | 171 | 0.1253 | <0.001 |
| Biomass | pH | Neutral (6.5–7.5) | 88 | 263.3 | 68.54 | 87 | 0.0372 | <0.001 |
| Biomass | pH | Slightly alkaline (7.6–8.5) | 87 | 835.86 | 90.03 | 86 | 0.0788 | <0.001 |
| NUE | pH | Overall | 310 | 2917.4 | 90 | 309 | 0.104 | <0.001 |
| NUE | pH | Acidic (<6.5) | 165 | 1941.56 | 92.03 | 164 | 0.1447 | <0.001 |
| NUE | pH | Neutral (6.5–7.5) | 61 | 250.8 | 79.81 | 60 | 0.0464 | <0.001 |
| NUE | pH | Slightly alkaline (7.6–8.5) | 84 | 693.27 | 86.04 | 83 | 0.0622 | <0.001 |

Where; MP = Micro plastics; Q = Cochran's heterogeneity statistic; I2 = percentage of total variation attributable to heterogeneity; DF = degrees of freedom; Tau2 = estimated between–study variance

**Table S9.** Overall heterogeneity statistics for the effects of MP/ Nano-plastic on root: shoot, biomass and NUE based on Cochran's Q test and $I^2$ statistics.

| Study Factor | Q | $I^2$ (%) | DF | Q-test p-value |
|---|---|---|---|---|
| Root: shoot ratio | 1324.800543 | 73.17106262 | 395 | <0.001 |
| Biomass | 3159.637783 | 88.50471235 | 378 | 0 |
| NUE | 3025.239132 | 89.77128249 | 327 | 0 |

Where; Q = Cochran's Q statistic; $I^2$ = percentage of total variability attributable to heterogeneity; DF = degrees of freedom.

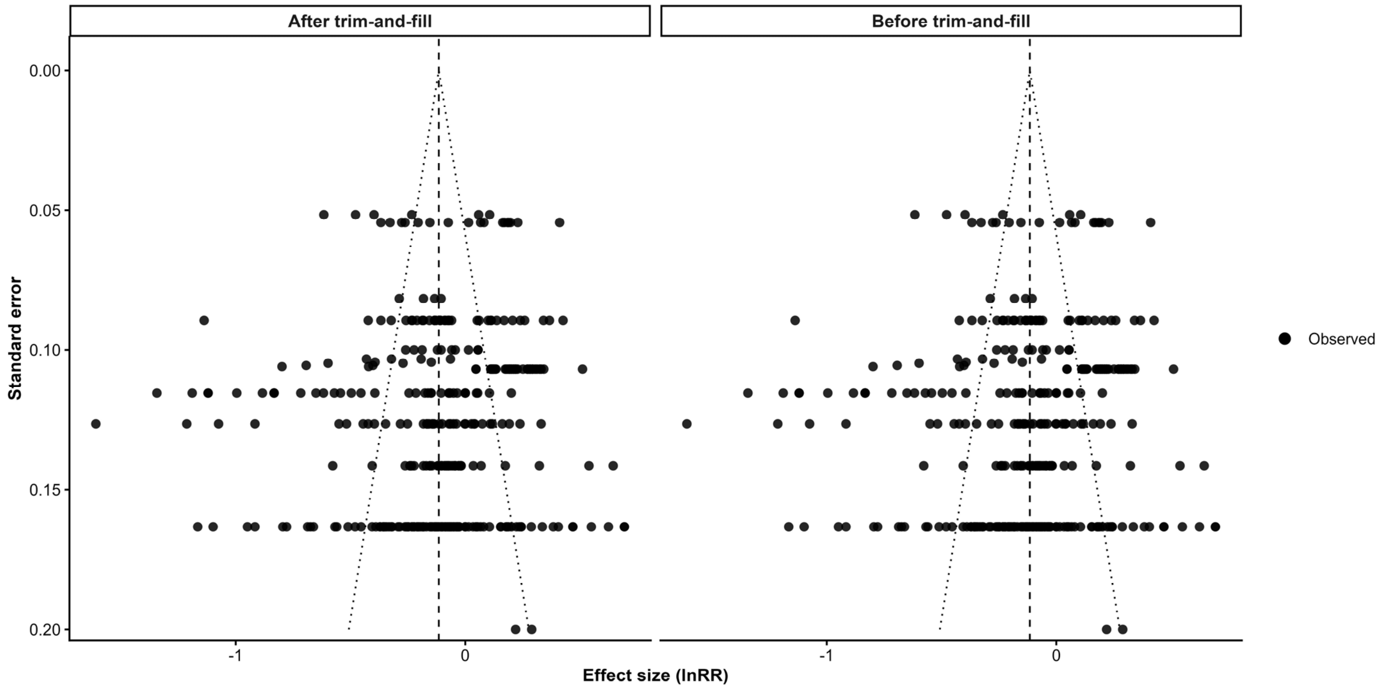


**Figure S1**. Funnel plot of effect size (InRR) of author publication bias before and after trim and fill.

## References

The list of the 50 published paper used in the data extraction for the meta–analysis study [1–50].


1. Guo, S.; Mu, L.; Sun, S.; Hou, X.; Yao, M.; Hu, X. Concurrence of Microplastics and Heat Waves Reduces Rice Yields and Disturbs the Agroecosystem Nitrogen Cycle. *J. Hazard. Mater.* **2023**, *452*, 131340, doi:10.1016/J.JHAZMAT.2023.131340.
2. Zhou, W.; Wang, Q.; Wei, Z.; Jiang, J.; Deng, J. Effects of Microplastic Type on Growth and Physiology of Soil Crops: Implications for Farmland Yield and Food Quality. *Environmental Pollution* **2023**, *326*, 121512, doi:10.1016/J.ENVPOL.2023.121512.
3. Yang, C.; Gao, X. Impact of Microplastics from Polyethylene and Biodegradable Mulch Films on Rice (Oryza Sativa L.). *Science of The Total Environment* **2022**, *828*, 154579, doi:10.1016/J.SCITOTENV.2022.154579.
4. Yang, J.; Song, K.; Tu, C.; Li, L.; Feng, Y.; Li, R.; Xu, H.; Luo, Y. Distribution and Weathering Characteristics of Microplastics in Paddy Soils Following Long-Term Mulching: A Field Study in Southwest China. *Science of The Total Environment* **2023**, *858*, 159774, doi:10.1016/J.SCITOTENV.2022.159774.
5. Yi, Z.; Zhang, Z.; Chen, G.; Rengel, Z.; Sun, H. Microplastics Have Rice Cultivar-Dependent Impacts on Grain Yield and Quality, and Nitrogenous Gas Losses from Paddy, but Not on Soil Properties. *J. Hazard. Mater.* **2023**, *446*, 130672, doi:10.1016/J.JHAZMAT.2022.130672.
6. Qi, Y.; Yang, X.; Pelaez, A.M.; Huerta Lwanga, E.; Beriot, N.; Gertsen, H.; Garbeva, P.; Geissen, V. Macro- and Micro- Plastics in Soil-Plant System: Effects of Plastic Mulch Film Residues on Wheat (Triticum Aestivum) Growth. *Science of The Total Environment* **2018**, *645*, 1048–1056, doi:10.1016/J.SCITOTENV.2018.07.229.
7. Lian, J.; Wu, J.; Xiong, H.; Zeb, A.; Yang, T.; Su, X.; Su, L.; Liu, W. Impact of Polystyrene Nanoplastics (PSNPs) on Seed Germination and Seedling Growth of Wheat (Triticum Aestivum L.). *J. Hazard. Mater.* **2020**, *385*, 121620, doi:10.1016/J.JHAZMAT.2019.121620.
8. De Souza Machado, A.A.; Lau, C.W.; Kloas, W.; Bergmann, J.; Bachelier, J.B.; Faltin, E.; Becker, R.; Görlich, A.S.; Rillig, M.C. Microplastics Can Change Soil Properties and Affect Plant Performance. *Environ. Sci. Technol.* **2019**, *53*, 6044–6052, doi:10.1021/ACS.EST.9B01339.
9. Wang, L.; Liu, Y.; Kaur, M.; Yao, Z.; Chen, T.; Xu, M. Phytotoxic Effects of Polyethylene Microplastics on the Growth of Food Crops Soybean (Glycine Max) and Mung Bean (Vigna Radiata). *International Journal of Environmental Research and Public Health 2021, Vol. 18, Page 10629* **2021**, *18*, 10629, doi:10.3390/IJERPH182010629.
10. Zhang, Y.; Zhang, C.; Jiang, M.; Zhou, G. Bio-Effects of Bio-Based and Fossil-Based Microplastics: Case Study with Lettuce-Soil System. *Environmental Pollution* **2022**, *306*, 119395, doi:10.1016/J.ENVPOL.2022.119395.
11. Yar, S.; Ashraf, M.A.; Rasheed, R.; Farooq, U.; Hafeez, A.; Ali, S.; Shahid, M.; Sarker, P.K. Taurine Decreases Arsenic and Microplastic Toxicity in Broccoli (Brassica Oleracea L.) through Functional and Microstructural Alterations. *BioMetals 2025 38:2* **2025**, *38*, 597–621, doi:10.1007/S10534-025-00667-9.
12. Debab, A.; Boudjabi, S.; Chenchouni, H.; Ababsa, N.; Brahimi, A. Effects of Incorporating Biochar on Soil Quality and Barley Yield in Microplastics-Contaminated Soils. *Chemosphere* **2024**, *368*, 143760, doi:10.1016/J.CHEMOSPHERE.2024.143760.
13. Gong, W.; Zhang, W.; Jiang, M.; Li, S.; Liang, G.; Bu, Q.; Xu, L.; Zhu, H.; Lu, A. Species-Dependent Response of Food Crops to Polystyrene Nanoplastics and Microplastics. *Science of The Total Environment* **2021**, *796*, 148750, doi:10.1016/J.SCITOTENV.2021.148750.
14. Iqbal, S.; Li, Y.; Xu, J.; Worthy, F.R.; Gui, H.; Faraj, T.K.; Jones, D.L.; Bu, D. Smallest Microplastics Intensify Maize Yield Decline, Soil Processes and Consequent Global Warming Potential. *J. Hazard. Mater.* **2025**, *486*, 136993, doi:10.1016/J.JHAZMAT.2024.136993.
15. Li, B.; Huang, S.; Wang, H.; Liu, M.; Xue, S.; Tang, D.; Cheng, W.; Fan, T.; Yang, X. Effects of Plastic Particles on Germination and Growth of Soybean (Glycine Max): A Pot Experiment under Field Condition. *Environmental Pollution* **2021**, *272*, 116418, doi:10.1016/J.ENVPOL.2020.116418.

16. Meng, F.; Yang, X.; Riksen, M.; Xu, M.; Geissen, V. Response of Common Bean (Phaseolus Vulgaris L.) Growth to Soil Contaminated with Microplastics. *Science of The Total Environment* **2021**, *755*, 142516, doi:10.1016/J.SCITOTENV.2020.142516.
17. Xia, H.; Shen, J.; Riaz, M.; Ran, F.; Cheng, T.; Wang, X.; Dong, Q.; Zu, C.; Liu, B.; Babar, S.; et al. Effects of Agricultural Plastic Films on Crop Growth and Soil Health in Tobacco Fields: A Comparative Study. *Applied Soil Ecology* **2025**, *206*, 105795, doi:10.1016/J.APSOIL.2024.105795.
18. Hu, Q.; Li, X.; Gonçalves, J.M.; Shi, H.; Tian, T.; Chen, N. Effects of Residual Plastic-Film Mulch on Field Corn Growth and Productivity. *Science of The Total Environment* **2020**, *729*, 138901, doi:10.1016/J.SCITOTENV.2020.138901.
19. Hu, C.; Wang, X.; Wang, S.; Lu, B.; Guo, W.; Liu, C.; Tang, X. Impact of Agricultural Residual Plastic Film on the Growth and Yield of Drip-Irrigated Cotton in Arid Region of Xinjiang, China. *International Journal of Agricultural and Biological Engineering* **2020**, *13*, 160–169, doi:10.25165/J.IJABE.20201301.5084.
20. Zhang, G.; Zhang, S.; Xia, Z.; Wu, M.; Bai, J.; Lu, H. Effects of Biodegradable Film and Polyethylene Film Residues on Soil Moisture and Maize Productivity in Dryland. *Agriculture (Switzerland)* **2023**, *13*, 332, doi:10.3390/AGRICULTURE13020332/S1.
21. Li, Z.; Li, R.; Li, Q.; Zhou, J.; Wang, G. Physiological Response of Cucumber (Cucumis Sativus L.) Leaves to Polystyrene Nanoplastics Pollution. *Chemosphere* **2020**, *255*, 127041, doi:10.1016/J.CHEMOSPHERE.2020.127041.
22. Das, E.J.; Alam, A.K.M.R. Effects of Microplastics Polluted Soil on the Growth of Solanum Lycopersicum L. *Environmental Systems Research 2024 13:1* **2024**, *13*, 36-, doi:10.1186/S40068-024-00367-2.
23. Boots, B.; Russell, C.W.; Green, D.S. Effects of Microplastics in Soil Ecosystems: Above and below Ground. *Environ. Sci. Technol.* **2019**, *53*, 11496–11506, doi:10.1021/ACS.EST.9B03304.
24. Dong, Y.; Gao, M.; Qiu, W.; Song, Z. Uptake of Microplastics by Carrots in Presence of As (III): Combined Toxic Effects. *J. Hazard. Mater.* **2021**, *411*, 125055, doi:10.1016/J.JHAZMAT.2021.125055.
25. Esterhuizen, M.; Kim, Y.J. Effects of Polypropylene, Polyvinyl Chloride, Polyethylene Terephthalate, Polyurethane, High-Density Polyethylene, and Polystyrene Microplastic on Nelumbo Nucifera (Lotus) in Water and Sediment. *Environmental Science and Pollution Research 2021 29:12* **2021**, *29*, 17580–17590, doi:10.1007/S11356-021-17033-0.
26. Lozano, Y.M.; Lehnert, T.; Linck, L.T.; Lehmann, A.; Rillig, M.C. Microplastic Shape, Polymer Type, and Concentration Affect Soil Properties and Plant Biomass. *Front. Plant Sci.* **2021**, *12*, 616645, doi:10.3389/FPLS.2021.616645/TEXT.
27. Jiang, X.; Chen, H.; Liao, Y.; Ye, Z.; Li, M.; Klobučar, G. Ecotoxicity and Genotoxicity of Polystyrene Microplastics on Higher Plant Vicia Faba. *Environmental Pollution* **2019**, *250*, 831–838, doi:10.1016/J.ENVPOL.2019.04.055.
28. Graf, M.; Greenfield, L.M.; Reay, M.K.; Bargiela, R.; Williams, G.B.; Onyije, C.; Lloyd, C.E.M.; Bull, I.D.; Evershed, R.P.; Golyshin, P.N.; et al. Increasing Concentration of Pure Micro- and Macro-LDPE and PP Plastic Negatively Affect Crop Biomass, Nutrient Cycling, and Microbial Biomass. *J. Hazard. Mater.* **2023**, *458*, 131932, doi:10.1016/J.JHAZMAT.2023.131932.
29. Saha, A.; Baruah, P.; Handique, S. Assessment of Microplastic Pollution on Soil Health and Crop Responses: Insights from Dose-Dependent Pot Experiments. *Applied Soil Ecology* **2024**, *203*, 105648, doi:10.1016/J.APSOIL.2024.105648.
30. Bosker, T.; Bouwman, L.J.; Brun, N.R.; Behrens, P.; Vijver, M.G. Microplastics Accumulate on Pores in Seed Capsule and Delay Germination and Root Growth of the Terrestrial Vascular Plant Lepidium Sativum. *Chemosphere* **2019**, *226*, 774–781, doi:10.1016/J.CHEMOSPHERE.2019.03.163.
31. De Silva, Y.S.K.; Rajagopalan, U.M.; Kadono, H.; Li, D. Effects of Microplastics on Lentil (Lens Culinaris) Seed Germination and Seedling Growth. *Chemosphere* **2022**, *303*, 135162, doi:10.1016/J.CHEMOSPHERE.2022.135162.
32. Pflugmacher, S.; Tallinen, S.; Kim, Y.J.; Kim, S.; Esterhuizen, M. Ageing Affects Microplastic Toxicity over Time: Effects of Aged Polycarbonate on Germination, Growth, and Oxidative Stress of Lepidium Sativum. *Science of The Total Environment* **2021**, *790*, 148166, doi:10.1016/J.SCITOTENV.2021.148166.
33. Urbina, M.A.; Correa, F.; Aburto, F.; Ferrio, J.P. Adsorption of Polyethylene Microbeads and Physiological Effects on Hydroponic Maize. *Science of The Total Environment* **2020**, *741*, 140216, doi:10.1016/J.SCITOTENV.2020.140216.

34. Wu, X.; Liu, Y.; Yin, S.; Xiao, K.; Xiong, Q.; Bian, S.; Liang, S.; Hou, H.; Hu, J.; Yang, J. Metabolomics Revealing the Response of Rice (Oryza Sativa L.) Exposed to Polystyrene Microplastics. *Environmental Pollution* **2020**, *266*, 115159, doi:10.1016/J.ENVPOL.2020.115159.
35. Dainelli, M.; Pignattelli, S.; Bazihizina, N.; Falsini, S.; Papini, A.; Baccelli, I.; Mancuso, S.; Coppi, A.; Castellani, M.B.; Colzi, I.; et al. Can Microplastics Threaten Plant Productivity and Fruit Quality? Insights from Micro-Tom and Micro-PET/PVC. *Science of The Total Environment* **2023**, *895*, 165119, doi:10.1016/J.SCITOTENV.2023.165119.
36. Bouaicha, O.; Tiziani, R.; Maver, M.; Lucini, L.; Miras-Moreno, B.; Zhang, L.; Trevisan, M.; Cesco, S.; Borruso, L.; Mimmo, T. Plant Species-Specific Impact of Polyethylene Microspheres on Seedling Growth and the Metabolome. *Science of The Total Environment* **2022**, *840*, 156678, doi:10.1016/J.SCITOTENV.2022.156678.
37. Lahiani, M.H.; Dervishi, E.; Chen, J.; Nima, Z.; Gaume, A.; Biris, A.S.; Khodakovskaya, M. V. Impact of Carbon Nanotube Exposure to Seeds of Valuable Crops. *ACS Appl. Mater. Interfaces* **2013**, *5*, 7965–7973, doi:10.1021/AM402052X.
38. Chen, S.; Feng, Y.; Han, L.; Li, D.; Feng, Y.; Jeyakumar, P.; Sun, H.; Shi, W.; Wang, H. Responses of Rice (Oryza Sativa L.) Plant Growth, Grain Yield and Quality, and Soil Properties to the Microplastic Occurrence in Paddy Soil. *J. Soils Sediments* **2022**, *22*, 2174–2183, doi:10.1007/S11368-022-03232-W.
39. Khan, M.A.; Kumar, S.; Wang, Q.; Wang, M.; Fahad, S.; Nizamani, M.M.; Chang, K.; Khan, S.; Huang, Q.; Zhu, G. Influence of Polyvinyl Chloride Microplastic on Chromium Uptake and Toxicity in Sweet Potato. *Ecotoxicol. Environ. Saf.* **2023**, *251*, 114526, doi:10.1016/J.ECOENV.2023.114526.
40. Pinto-Poblete, A.; Retamal-Salgado, J.; Zapata, N.; Sierra-Almeida, A.; Schoebitz, M. Impact of Polyethylene Microplastics and Copper Nanoparticles: Responses of Soil Microbiological Properties and Strawberry Growth. *Applied Soil Ecology* **2023**, *184*, 104773, doi:10.1016/J.APSOIL.2022.104773.
41. Wang, J.; Li, J.; Liu, W.; Zeb, A.; Wang, Q.; Zheng, Z.; Shi, R.; Lian, Y.; Liu, L. Three Typical Microplastics Affect the Germination and Growth of Amaranth (Amaranthus Mangostanus L.) Seedlings. *Plant Physiology and Biochemistry* **2023**, *194*, 589–599, doi:10.1016/J.PLAPHY.2022.12.007.
42. Yang, R.; Cui, Y.; Cheng, L.; Hu, M.; Guan, M.; Fu, Y.; Zhang, Y. Exposure to Polyethylene Terephthalate Microplastics Induces Reprogramming of Flavonoids Metabolism and Gene Regulatory Networks in Capsicum Annuum. *Ecotoxicol. Environ. Saf.* **2025**, *293*, 118022, doi:10.1016/J.ECOENV.2025.118022.
43. Weerasinghe, W.M.T.M.; Madawala, H.M.S.P. Potential Impacts of Two Types of Microplastics on Solanum Lycopersicum L. and Arbuscular Mycorrhizal Fungi. *Ceylon Journal of Science 51*, 137–146, doi:10.4038/cjs.v51i2.8008.
44. Kanold, E.; Buchanan, S.W.; Dunfield, K.; Antunes, P.M. Microplastic Additions Modulate Intraspecific Variability in Root Traits and Mycorrhizal Responses across Root-Life History Strategies. *Funct. Ecol.* **2024**, *38*, 2369–2377, doi:10.1111/1365-2435.14659;WEBSITE:WEBSITE:BESJOURNALS;REQUESTEDJOURNAL:JOURNAL:13652435;WGROUP:STRING:PUBLICATION.
45. Pignattelli, S.; Broccoli, A.; Renzi, M. Physiological Responses of Garden Cress (L. Sativum) to Different Types of Microplastics. *Science of The Total Environment* **2020**, *727*, 138609, doi:10.1016/J.SCITOTENV.2020.138609.
46. Sahasa, R.G.K.; Dhevagi, P.; Poornima, R.; Ramya, A.; Moorthy, P.S.; Alagirisamy, B.; Karthikeyan, S. Effect of Polyethylene Microplastics on Seed Germination of Blackgram (Vigna Mungo L.) and Tomato (Solanum Lycopersicum L.). *Environmental Advances* **2023**, *11*, 100349, doi:10.1016/J.ENVADV.2023.100349.
47. Xu, N.; Song, Y.; Zheng, C.; Li, S.; Yang, Z.; Jiang, M. Indole-3-Acetic Acid and Zinc Synergistically Mitigate Positively Charged Nanoplastic-Induced Damage in Rice. *J. Hazard. Mater.* **2023**, *455*, 131637, doi:10.1016/J.JHAZMAT.2023.131637.
48. Ren, J.; Luo, X.; Luo, Y.; Xu, X.; Wang, Q.; Zhang, Q. Interactive Effects of Combined Manganese and Microplastic Pollution on Soybean Growth. *Front. Plant Sci.* **2025**, *16*, 1699129, doi:10.3389/FPLS.2025.1699129/TEXT.
49. Shi, L.; Chen, Z.; Hou, Y.; Li, J.; Shen, Z.; Chen, Y. The Original Polyethylene Microplastics Inhibit the Growth of Sweet Potatoes and Increase the Safety Risk of Cadmium. *Front. Plant Sci.* **2023**, *14*, 1138281, doi:10.3389/FPLS.2023.1138281/TEXT.

50. Ahmad, Z.; Najaf, D.; Atif, S.; Chaudhry, H.; Aslam, F.; Bhatti, S.G.; Alsaiari, N.S.; Al-Buriahi, M.S.; Sesay, M. Assessment of Physiological Stress on Plants Grown in Soil Contaminated with Microplastics. *Scientific Reports 2025 15:1* **2025**, *15*, 35551-, doi:10.1038/s41598-025-19610-6.

**Disclaimer/Publisher's Note:** The statements, opinions and data contained in all publications are solely those of the individual author(s) and contributor(s) and not of MDPI and/or the editor(s). MDPI and/or the editor(s) disclaim responsibility for any injury to people or property resulting from any ideas, methods, instructions or products referred to in the content.
